# Evolution, structure, and drug-metabolizing activity of mammalian prenylcysteine oxidases

**DOI:** 10.1016/j.jbc.2024.107810

**Published:** 2024-09-24

**Authors:** Marco Barone, Letizia Pizzorni, Marco W. Fraaije, Maria L. Mascotti, Andrea Mattevi

**Affiliations:** 1 Department of Biology and Biotechnology “Lazzaro Spallanzani”, University of Pavia, Pavia, Italy; 2 Molecular Enzymology Group, University of Groningen, Groningen, The Netherlands; 3 IHEM CONICET, Universidad Nacional de Cuyo, Mendoza, Argentina

**Keywords:** protein prenylation, enzyme evolution, flavin, drug metabolism, lipoprotein oxidation, enzyme inhibitor, oxidative metabolism

## Abstract

Prenylcysteine oxidases (PCYOXs) metabolize prenylated cysteines produced by protein degradation. They utilize oxygen as a co-substrate to produce free cysteine, an aldehyde, and hydrogen peroxide through the unusual oxidation of a thioether bond. In this study, we explore the evolution, structure, and mechanism of the two mammalian PCYOXs. A gene duplication event in jawed vertebrates originated in these two paralogs. Both enzymes are active on farnesyl- and geranylgeranylcysteine, but inactive on molecules with shorter prenyl groups. Kinetics experiments outline a mechanism where flavin reduction and re-oxidation occur rapidly without any detectable intermediates, with the overall reaction rate limited by product release. The experimentally determined three-dimensional structure of PCYOX1 reveals long and wide tunnels leading from the surface to the flavin. They allow the isoprene substrate to curl up within the protein and position its reactive cysteine group close to the flavin. A hydrophobic patch on the surface mediates membrane association, enabling direct substrate and product exchange with the lipid bilayer. Leveraging established knowledge of flavoenzyme inhibition, we designed sub-micromolar PCYOX inhibitors. Additionally, we discovered that PCYOXs bind and slowly degrade salisirab, an anti-RAS compound. This activity suggests potential and previously unknown roles of PCYOXs in drug metabolism.

Human proteins are frequently modified through the addition of a lipid tail (1). Among the various types of lipidations, prenylation is particularly common, targeting 2% of the total proteome. Prenyl groups, which include 15-carbon farnesyl and 20-carbon geranylgeranyl moieties, are synthesized from isoprenoid units (2, 3). The prenylation process comprises farnesyl and geranylgeranyl transferases, RAS-converting enzyme 1, isoprenylcysteine carboxyl methyltransferase, and phosphodiesterase δ. Inhibition of these enzymes has been extensively pursued as a strategy to disrupt the oncogenic function of RAS proteins (4, 5). Conversely, the metabolic fate of prenylated proteins has been less studied. In a landmark discovery in 1997, Casey’s group identified a novel enzyme capable of metabolizing prenylcysteine residues (6). Initially described as a lyase, it was later found to be an oxidase and renamed prenylcysteine oxidase 1 (PCYOX1) (7, 8). PCYOX1 contains a non-covalently bound flavin and metabolizes free prenylcysteine into cysteine, an isoprenoid aldehyde, and a stoichiometric amount of hydrogen peroxide (Fig. 1*A*). When absent, farnesylcysteine and geranylgeranylcysteine accumulate in the brain and liver (9). The proteomic analysis highlighted PCYOX1 as a major component of low-density lipoproteins (10). Its knockout was found to lower the levels of lipid peroxidation and protect from fatty accumulation in the walls of arteries (*e.g*. atheroprogression). These findings identify PCYOX1 as a potential drug target for evaluation against cardiovascular diseases (11). The human gene for PCYOX1 is located on chromosome 2, but a paralog, encoding a PCYOX1-like protein with a predicted prenylcysteine-binding domain and 43% of sequence identity to PCYOX1, is present on chromosome 5. Recent studies have shown that this protein is necessary for the bactericidal activity of neutrophils (12). Hence, although the PCYOX enzymes have been somewhat neglected in the context of mammalian metabolism, a growing body of evidence now supports their biological roles.

**Figure 1:**
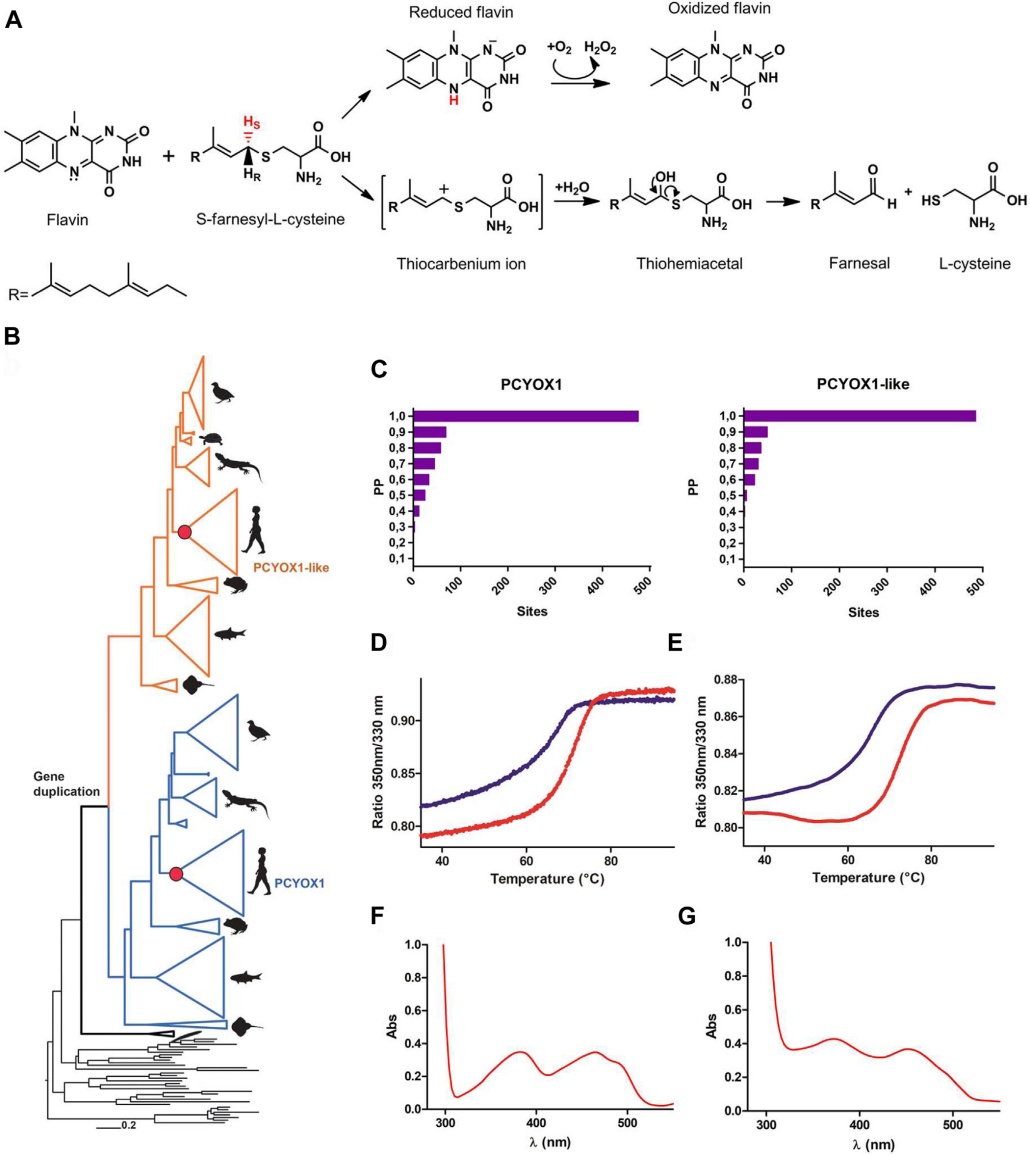
**PCYOX enzymes and their evolution.***A*, proposed mechanism of the oxidative thioether cleavage by PCYOX1. The reaction is initiated by the extraction of pro-S-hydride (H_s_), which leads to the formation of the reduced flavin and a thiocarbenium ion. The latter spontaneously hydrates, producing an unstable thiohemiacetal that rearranges into the corresponding C1-aldehyde and L-cysteine. Molecular oxygen is used as a co-substrate to re-oxidize FAD, generating hydrogen peroxide as a byproduct. *B*, condensed maximum likelihood tree of chordata PCYOXs (fully annotated phylogeny Fig. S1). Silhouettes (from https://www.phylopic.org/) indicate the subphyla/order: aves, testudines, squamata, mammals, amphibia, actinopterygii, chondrichthyes and cephalochordata. The ancestral PCYOXs experimentally characterized here are shown as red circles. Their amino acid sequences are listed in Fig. S2. The scale-bar indicates substitutions per site. *C*, Statistical confidence of mammalian ancestors of PCYOX1 (*left*) and PCYOX1-like (*right*). Residues subjected to post-translational modifications, N-glycosylation and phosphorylation are conserved and reconstructed with PPs equal to 1 (Fig. 3 and Table S1) (44, 45, 46). *D–E*, Thermal shift assays. The lines show the unfolding profile of 10 μM PCYOX (D) and 10 μM PCYOX1-like (*E*) in the absence (*blue*) and presence (*red*) of 100 μM S-farnesyl-L-cysteine. The unfolding temperatures are listed in Table 1. *F–G*, *UV-Vis* absorbance spectra of 40 μM PCYOX1 (*F*) and 40 μM PCYOX1-like (*G*).

Ancestral sequence reconstruction has proven to be a powerful approach to elucidate the biochemistry and structure of membrane-associated proteins (13). We therefore opted for this method in the investigation of PCYOXs as they are associated with the cell membranes, require detergents for their handling, and are relatively difficult to produce as recombinant proteins (7, 8). Specifically, we targeted the mammalian ancestors of both PCYOX1 and PCYOX1-like. We show that the two enzymes are redundant, structurally and functionally. Both act on farnesylcysteine and geranylgeranylcysteine, efficiently utilizing oxygen as a co-substrate. These findings provide insight into the evolution of PCYOXs and the mechanism underpinning their characteristic C-S bond oxidizing activity. By means of the experimental structure determination, we describe their substrate and membrane binding regions and the design of potent non-covalent inhibitors. Furthermore, we show that PCYOX1 is active on salirasib, an anti-RAS inhibitor, suggesting potential roles for PCYOXs in xenobiotic metabolism in addition to their established function in prenylcysteine elimination.

## Results

### PCYOXs gene duplication occurred in jawed vertebrates

To infer the phylogeny of PCYOXs, we gathered a dataset of homologs (295 sequences) from the metazoan kingdom. Subphylum and classes among Chordata phyla were more densely vetted. (Fig. 1*B*; Fig. S1). The homology search was conducted employing the human PCYOX sequences as queries on each phylum. The resulting phylogeny clearly indicates that PCYOX1 and PCYOX1-like clades arose from a gene duplication event from a common ancestor at the emergence of *Gnatosthomata* about 460 million years ago (14). The taxonomic distribution of both paralogs is the same, suggesting that both gene copies fulfill a functional role in jawed vertebrates.

By performing ancestral sequence reconstruction, we targeted the sequences of the mammalian ancestors of PCYOX1 and PCYOX1-like. Both were reconstructed with high posterior probabilities (0.93 and 0.96, respectively) (15) (Figs. 1*C* and Fig. S2; Table S1). As they show high sequence identities with their human counterparts (78% and 88%, respectively) they were selected for experimental characterization as models to understand the human proteins (Fig. S3). Hereafter, PCYOX1 and PCYOX1-like will refer to these two ancestors.

### PCYOX1-like and PCYOX1 share the same substrate profile

The N-terminal signaling peptide was not included for recombinant protein production and the PCYOX1 and PCYOX1-like ancestors used in this work correspond to the mature proteins comprising 475 and 472 amino acids, respectively. Both enzymes were expressed in *E. coli* (Fig. S4). PCYOX1 showed high expression yields, producing several milligrams of protein per liter of culture. It could be easily extracted from the membrane and purified. PCYOX1-like had lower expression levels and required mild extraction conditions from inclusion bodies (16). Both proteins retained non-covalently bound FAD and exhibited high >60 °C unfolding temperatures (Table 1; Fig. 1, *D*–*G*). Of note, attempts to express the human PCYOX enzymes in *E. coli* failed while its expression in HEK cells was minimal. We surmise that our strategy of using ancestral sequence reconstruction proved successful, providing experimentally tractable forms of both PCYOX1 and PCYOX1-like.

**Table 1:** Steady-state kinetics and thermal shifts

	Substrate	K_M_ (μM)	*k*_cat_ (min^−1^)	*k*_cat_/K_M_ (min^−1^ μM^−1^)	ΔT (°C)^a^
PCYOX1					
Ancestral	*S*-farnesyl-L-cysteine	5.07 ± 0.44	1.36 ± 0.03	0.27	+5.1
	*S*-geranylgeranyl-L-cysteine	9.79 ± 2.18	0.27 ± 0.02	0.028	+6.6
	*S*-geranyl-L-cysteine	-	no activity	-	+1.3
	*S*-allyl-L-cysteine	-	no activity	-	+0.5
	*S*-farnesyl-L-cysteine methyl ester	2.59 ± 0.34	0.38 ± 0.01	0.15	+0.7
	*N*-acetyl-*S*-farnesyl-L-cysteine	69.3 ± 13.6	1.28 ± 0.10	0.018	+4.4
	*S*-farnesyl-thiosalicylic acid^b^	>37	>0.18	> 0.005	+2.2
	farnesyl-*N*-tranylcypromine	-	inhibitor^c^	-	+4.7
	farnesyl-*S*-propargyl	-	inhibitor^d^	-	+1.3
	farnesyl-*S*-fluorene	-	-	-	+0.9
Y433A	*S*-farnesyl-L-cysteine	24.4 ± 5.0	0.64 ± 0.05	0.026	+2.2
	farnesyl-*N*-tranylcypromine	-	inhibitor	-	+7.7
V234W	*S*-farnesyl-L-cysteine	-	no activity	-	+6.2
	farnesyl-*N*-tranylcypromine	-	-	-	+11.8
Y221A	*S*-farnesyl-L-cysteine	-	no activity	-	+0.1
	farnesyl-*N*-tranylcypromine	-	-	-	−0.7
Human^e^	*S*-farnesyl-L-cysteine	3.0 ± 0.7	0.48 ± 0.03	0.16	-
PCYOX1-like					
Ancestral	*S*-farnesyl-L-cysteine	11.5 ± 3.5	1.7 ± 0.1	0.15	+6.1
	*S*-geranylgeranyl-L-cysteine	7.1 ± 2.0	0.28 ± 0.03	0.039	+11.4
	*S*-geranyl-L-cysteine	-	no activity	-	+0.9

a Difference with the unfolding temperature of the proteins without ligand (67.4 °C for PCYOX1 and 66.3 °C for PCYOX1-like).

b S-farnesyl-thiosalicylic acid is salirasib. Due to its poor solubility, we were unable to reach saturation (Fig. S5*H*) and only an estimate of the K_M_ and K_cat_ values could be obtained.

c IC_50_ = 2.2 ± 1.1 μM (Fig. S8*B*); K_i_=613 ± 95 nM (Fig. S8*D*).

d IC_50_ = 5.1 ± 1.2 μM (Fig. S8*C*); K_i_=4.8 ± 0.4 μM (Fig. S8*E*).

e Data are from (18).

Both PCYOX1 and PCYOX1-like displayed activity on the long-chain substrates, *S*-farnesyl-L-cysteine (15-carbon isoprenoid) and *S*-geranylgeranyl-L-cysteine (20-carbon) whereas no conversion was detected using shorter *S-*geranyl-L-cysteine (10-carbon) or *S-*allyl-L-cysteine (3-carbon) (Table 1; Fig. S5, *A–D*). The *k*_*cat*_ and K_M_ values are similar for the two enzymes and agree with the published values measured for the human PCYOX1 expressed in insect cells (Table 1) (8). These findings are critical as they demonstrate that PCYOX1-like is a second mammalian prenylcysteine oxidase. Like PCYOX1, it acts on long-chain isoprenoid substrates with a preference for *S-*farnesyl-L-cysteine. These properties differ from the enzymatic systems of plants where the single PCYOX enzyme copy acts on geranyl compounds (17).

Taking advantage of the high expression yields of PCYOX1, we further investigated its substrate specificity using modified compounds with substituents at the cysteine carboxyl (*S-*farnesyl-L-cysteine methyl ester) and amino (*N-*acetyl-*S-*farnesyl-L-cysteine) groups. Esterification of the carboxyl group had little effect on enzyme efficiency, whereas amine acetylation significantly reduced enzyme activity, decreasing the efficiency by 10-fold (Table 1; Fig. S5, *E–F*). This indicates that the free α−amino group is crucial for substrate recognition and confirms that PCYOXs are not intended to metabolize prenylated proteins (8).

### Pre-steady state kinetics outlines fast reactivities and slow product release

**Figure 2:**
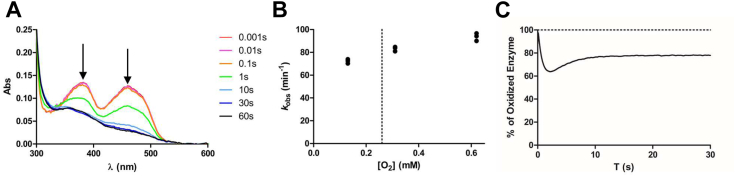
**Pre-Steady state kinetics of PYCOX1.***A*, enzyme (12.5 μM) reduction upon anaerobic mixing with 20-fold excess of *S*-farnesyl-L-cysteine. Arrows represent the absorbance peaks of the oxidized flavin. *B*, observed rates for flavin re-oxidation after mixing reduced enzyme (12.5 μM) with different oxygen concentrations at 25 °C. The dotted line represents the observed *k*_ox_ (83.4 min^−1^) at atmospheric concentration of dioxygen (0.26 mM). *C*, Percentage of oxidized flavin upon aerobic mixing of the enzyme with of *S*-farnesyl-L-cysteine (20-fold excess). After transiently becoming reduced by about 40%, the enzyme remains largely oxidized (78%) during the steady-state kinetic phase.

To dissect the individual catalytic steps, we employed stopped-flow kinetics using PCYOX1 and *S-*farnesyl-L-cysteine as the reference system. The reductive half-reaction was analyzed by anaerobically following the flavin redox state after the addition of an excess of substrate, while the oxidative half-reaction was traced by monitoring the re-oxidation of the reduced enzyme upon exposure to different oxygen concentrations. Using a relatively high concentration of *S-*farnesyl-L-cysteine (250 μM) we measured a rate of 118.7 min^−1^ for the reductive (*k*_red_) half-reaction (Fig. 2*A*; Fig. S9*C*). Reoxidation at atmospheric oxygen concentration was relatively fast: 83.4 min^−1^. Interestingly, the re-oxidation of the reduced enzyme showed little dependency on oxygen concentration within the range used for the experiment (0.13–0.62 mM, Fig. 2*B*). No transient intermediates, such as substrate-flavin or oxygen-flavin adducts or charge-transfer complexes, were observed in the reductive and oxidative-half reactions. With rates in the order of 80 to 120 min^−1^, flavin reduction and re-oxidation are faster than the overall turnover number (*k*_cat_ = 1.36 min^−1^; Table 1), consistent with the notion that the rate-limiting step of the overall reaction is product release (18). Supporting this interpretation, the enzyme is mostly in the oxidized state during turnover (Fig. 2*C*). We conclude that PCYOX1 is rapidly reduced by the substrate, likely through hydride transfer (Fig. 1*A*). This is followed by the efficient reaction of the reduced flavin with oxygen to release hydrogen peroxide. We assume that after its oxidation, the substrate forms a thiocarbenium ion that undergoes spontaneous hydrolysis, potentially inside the active site. Finally, the release of the bulky farnesal aldehyde is the slowest event in the process.

### Extended cavities for poly-isoprenoid binding

**Figure 3:**
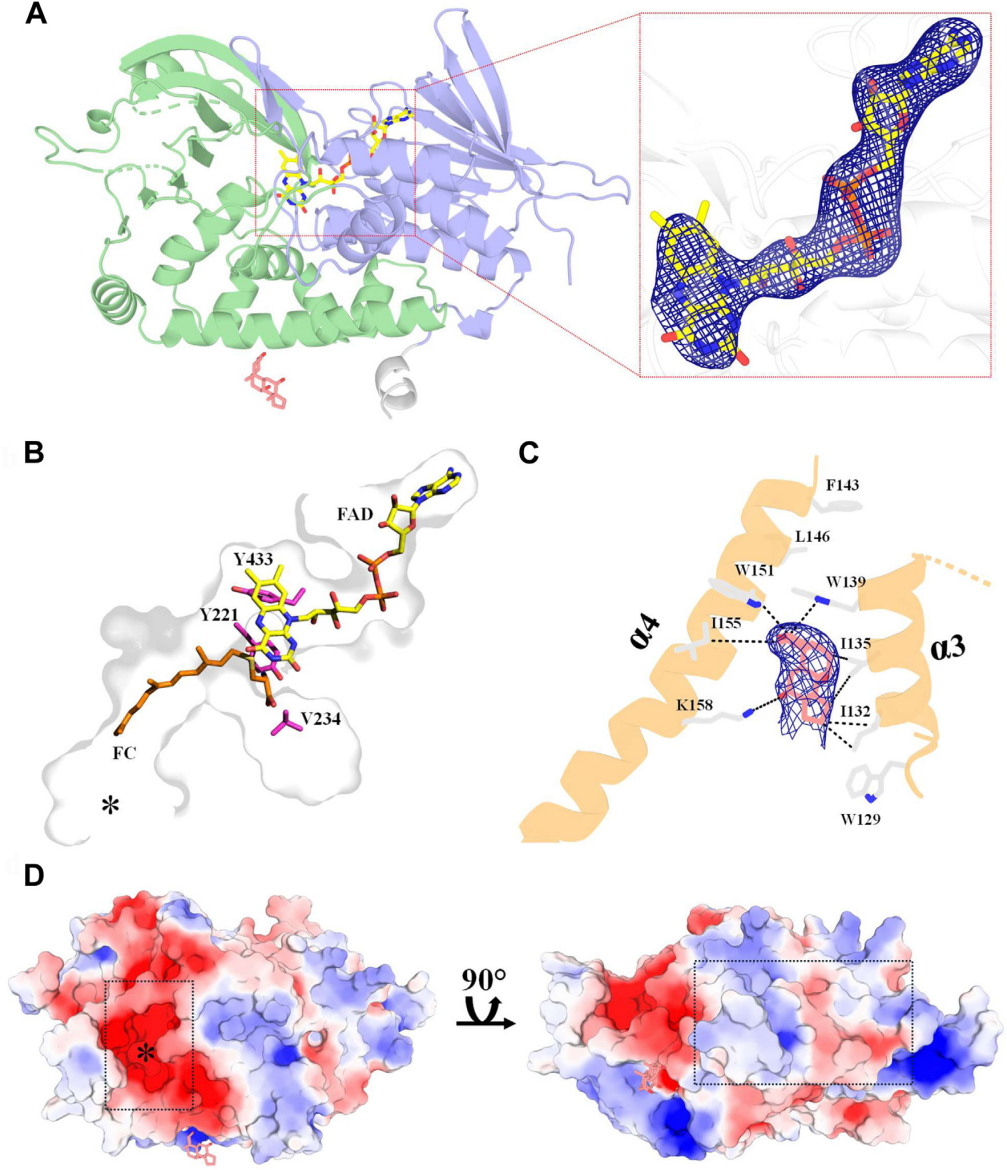
**Crystal Structure of PCYOX1.***A*, overall structure at 3.1 Å resolution. The FAD- and substrate-domains are in *violet* and *green*, respectively. FAD and CHAPS carbons are in *yellow* and *pink*. The inset depicts the FAD polder omit map calculated by excluding the ligand and countered at 3.5 σ. *B*, DiffDOCK (47) predicted binding of *S*-farnesyl-L-cysteine and residues targeted by mutagenesis. The asterisk marks the entrance of the active site tunnel. *C*, hydrophobic interactions of PCYOX1 and CHAPS in crystal packing, α4, and α3 belong to two adjacent protein molecules. The polder omit map was calculated by excluding the ligands and contoured at 3.5 σ. *D*, the electrostatic potential mapped onto the accessible surface area of PCYOX1. The dotted rectangle outlines the entrance of the active site (on the *left*, asterisk) and the hydrophobic portion on the underside of PCYOX1 that may promote membrane association (on the *right*).

Reflecting its high stability and operability, PCYOX1 could be crystallized, and its structure was solved at 3.1 Å resolution using AlphaFold2-predicted coordinates as a search model (Table S2). Despite the medium resolution, the quality of the electron density maps was sufficient to analyze the main structural features (Fig. S6). PCYOX1, a monomeric protein, is composed of two domains: the classical dinucleotide-binding domain with the Rossmann motif and a specific prenylcysteine-oxidase domain. The FAD prosthetic group is bound to the Rossmann fold in an extended conformation (Fig. 3*A*). This architecture generally resembles that of amino acid and amine oxidases (19), such as (S)-6-hydroxynicotine oxidase from *Shinella* sp HZN7 (PDB ID 6CR0; 15% sequence identity, root-mean-square deviation of 3.1 Å for 364 Cα atoms) and putrescine oxidase from *Rhodococcus erythropolis* (2YG7, 14% sequence identity, root-mean-square deviation of 3.6 Å for 378 Cα atoms). Beside this overall similarity, PCYOX1 is distinguished by the presence of a wide and elongated tunnel-shaped cavity extending orthogonally from the flavin ring to the protein surface (Fig. 3*B*). Computational docking predicts that the substrate binds with the isoprenoid tail extended along the cavity towards its entrance (Fig. 3*B*). Although lacking sufficient detail for mechanistic considerations, the computationally modeled binding mode is compatible with catalysis, as it positions the cysteine-isoprenoid thioether bond directly in front of the flavin to undergo oxidative cleavage. With its wide shape, the cavity in the interior of the protein can provide ample space also for oxygen diffusion (Fig. 1*A*).

To validate our structural analysis, we generated mutants targeting three amino acids located at the inner segment of the above-described tunnels characterizing the active site core (Fig. 3*B*). Tyr433 (PP=1), replaced by histidine in PCYOX1-like (PP=1), is predicted to interact with the cysteine moiety of the substrate at the bottom of the substrate cavity and in direct contact with the flavin. The nearby Tyr221 is conserved in both PCYOX1 (PP=1) and PCYOX1-like (PP=1) and could also directly or indirectly interact with the substrate. Mutating these two tyrosine residues to alanine was therefore expected to remove bulky side chains involved in substrate binding. Val234 (PP=1; methionine in PCYOX1-like, PP=0.99) was chosen as it is well positioned to create steric hindrance in the substrate tunnel through its replacement with tryptophan. These three sites were unambiguously reconstructed, as evidenced by their high posterior probabilities, and they also showed conservation among the extant and ancestral PCYOX1, suggesting a potential role in catalysis or protein stability. Y433A substitution had little effect as the mutant enzyme retained the enzymatic activity (Fig. S5*G*). In contrast, Y221A was devoid of any measurable activity confirming that this side chain likely interacts, directly or through water-mediated hydrogen bonds, with the cysteine polar head of the substrate (Fig. 3*B*). Consistently, *S-*farnesyl-L-cysteine did not cause any protein stabilisation suggesting that substrate binding is severely compromised by this mutation (Table 1). Also, V434W was inactive though with no apparent loss of substrate binding as gathered by the thermal shift analysis (Table 1). We infer that the presence of bulky tryptophan compromises the attainment of the catalytically competent conformation of the bound substrate. Collectively, the mutagenesis experiments substantiated the idea that the bulky ligands are accommodated, in their entire length, within the large cavity characterizing PCYOX1. With its hydrogen bonding groups, the cysteine moiety is critical for finely positioning the thioether bond close to the flavin.

The structure of PCYOX1 does not display a well-defined membrane binding subdomain. However, a large hydrophobic patch is located on the underside of the enzyme. An electron density peak on this surface region convincingly matches the shape of the sterolic head of CHAPS, the detergent used for crystallization (Fig. 3*C*). This region likely represents the site of association of PCYOX1 to the membrane and may act synergistically with the KTEL C-terminal sequence to retain the protein in the endoplasmic reticulum (Fig. 3*D*) (11, 20). Notably, the entrance to the substrate binding cavity is adjacent to this hydrophobic patch. We speculate that these structural features might facilitate the direct diffusion of the highly hydrophobic substrates and products from and to the lipid bilayer. This mode of operation is likely shared by PCYOX1-like, as its AlphaFold3-predicted model outlines a highly similar substrate cavity and hydrophobic patch (Fig. S7, *A–B*) (21).

### Inhibitor discovery by design

PCYOX1 inhibition may be beneficial for mitigating the H_2_O_2_-triggered oxidation of phospholipids within lipoproteins, potentially opening new therapeutic avenues for cardiovascular diseases like atherosclerosis (11). Enzyme inhibitors can also serve as valuable tools for interrogating the roles of prenylcysteine metabolism in cell and tissue physiology (12). In this regard, the tunnel-shaped substrate site featured by PCYOX offered a clue by outlining the poly-isoprene moiety as a promising initial scaffold for binding. We then took advantage of the extensive knowledge of flavoenzyme inhibition and designed three molecules that combine a farnesyl group with chemical warheads found in various classes of covalent inhibitors targeting flavin-dependent oxidases, such as monoamine oxidases (Fig. S8*A*) (22, 23). Their binding and inhibitory properties were assessed by thermal shift and activity assays (Table 1). Two compounds, farnesyl-*S-*propargyl and farnesyl-*N-*tranylcypromine, proved to be strong inhibitors with single-digit micromolar IC_50_ values (Fig. S8, *B–C*). Neither of them modified the flavin spectrum, implying that they do not react with the flavin but rather function as non-covalent inhibitors. Further kinetic analysis indicated that farnesyl-*N-*tranylcypromine inhibits PCYOX1 competitively with a K_i_ value of 613 nM (Fig. S8*D*), while farnesyl-S-propargyl is a non-competitive inhibitor with a K_i_ of 4.8 μM (Fig. S8*E*). We speculate that the different types of inhibition might reflect different binding positions inside the tunnel due to their largely different substituents (propargyl *vs* tranylcypromine) attached to the farnesyl group. These first PCYOX inhibitors verify that the enzyme active site is tailored for binding ligands comprising two or three isoprene units and represent promising leads for further inhibitor development.

### A potential role in drug-metabolism

**Figure 4:**
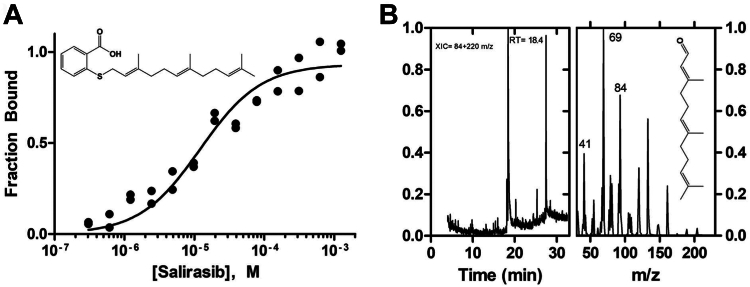
**Drug metabolism of PCYOX1.***A*, salirasib binding was measured with microscale thermophoresis (K_d_=12 ± 0.2 μM). *B*, extracted Ion Chromatogram (XIC) at 84 + 220 m/z after overnight incubation (30 °C) of 10 μM PCYOX1 with 1 mM salirasib together with the associated mass spectra at retention time of 18.4 min. Farnesal (220 m/z) detection was performed through a comparison with the NIST database. 84 m/z is a specific fragment of the farnesal prenyl chain. Intensities are normalized.

Inhibitors of protein farnesylation have been extensively sought as tools to disrupt the activity of oncogenic RAS (5). These compounds often comprise a poly-isoprene scaffold that mimics farnesol and thus could well bind to the active site of PCYOXs. We considered S-farnesyl-thiosalicylic acid (salirasib) because it reached clinical evaluation and has been extensively used to probe RAS signaling pathways (24). Salirasib harbors a thiosalicylate ring replacing the cysteine moiety of the substrate *S-*farnesyl-L-cysteine (Fig. 4*A*). Thermal shift and microscale thermophoresis assays indicated that the drug binds to PCYOX1 with a K_d_ value of 12 μM (Fig. 4*A*). Most interestingly, we found that the enzyme slowly metabolizes the drug, generating farnesal, the product of the oxidative cleavage of the thioether bond (Fig. 4*B*; Table 1; Fig. S5*H*). These findings indicate that PCYOXs might have a previously unappreciated role in drug binding and metabolism. With their large and hydrophobic binding cavities, these enzymes should be considered as potential binders and, if chemically feasible, metabolizers of 15- or 20-carbon polyisoprene-containing compounds.

## Discussion

Our work demonstrates that PCYOX1-like is a prenylcysteine oxidase with kinetic properties similar to those of PCYOX1. Both enzymes catalyze thioether bond cleavage through an oxidative process, are active on *S-*farnesyl-L-cysteine and *S-*geranylgeranyl-L-cysteine, do not metabolize shorter prenyl chain compounds, and use oxygen as an electron acceptor. Rapid kinetics experiments on PCYOX1s show that the substrate is rapidly oxidized without forming spectroscopically identifiable intermediates. The release of the hydrophobic reaction products limits the overall reaction rate, and the bulky *S-*geranylgeranyl-L-cysteine is the slowest substrate (Table 1). These kinetic features can be rationalized through the inspection of the experimental structure of PCYOX1 and its comparison with the AlphaFold3-predicted model of PCYOX1-like. The submerged substrate binds in elongated tunnels inside the protein and positions its C-S bond in contact with the flavin (Fig. 3*B*). As often observed for enzymes operating at the membrane surface (25), the substrate likely diffuses from the membrane into the active site. This raises questions about the fate of the farnesal product, a potentially toxic oxidant that can react with protein cysteine residues, effectively functioning as a “chemical prenylator” (26). The structure suggests that it might be released to the bilayer and become available for metabolic recycling, though the exact process and site of its metabolism remain open for further research (27).

Although unable to react with the flavin, our designed farnesyl-tranylcypromine compound is an effective, competitive inhibitor of PCYOX1, likely exploiting the substrate site for binding. Such a sub-micromolar affinity highlights PCYOXs as potential binders of prenylated metabolites. A compelling demonstration of this concept is our discovery that PCYOXs can metabolize salirasib, a widely studied drug that interferes with prenylated RAS activities. We suggest that PCYOXs should be included in the “avoidome,” the proteins determining the metabolism and pharmacokinetic properties of drugs, often producing undesirable effects (28). Simultaneously, the potential for drug interaction and inhibition opens avenues for pharmacological applications targeting these enzymes.

Our phylogenetic analysis indicates that the divergence between PCYOX1 and PCYOX1-like aligns with the divergence of jawed vertebrates, implying an increased PCYOX dosage in the gnathostomes. According to the Human Protein Atlas (29), PCYOX1 and PCYOX1-like have different subcellular localizations (30): PCYOX1 is found in the endoplasmic reticulum and is abundant in lipoproteins and blood, while PCYOX1-like is generally less expressed and primarily located in the nucleoplasm and mitochondria. This indicates that these enzymes might have complementary roles in vertebrates’ physiology. The increase in protein prenylation throughout animal evolution (31) likely exerted pressure to duplicate PCYOX, accommodating the greater production of prenylcysteine from the degradation and removal of prenylated proteins in various tissues and cellular organelles.

## Experimental procedures

### Chemicals

S-farnesyl-L-cysteine, S-geranyl-L-cysteine were from Avanti Polar Lipids; S-geranylgeranyl-L-cysteine from MuseChem; S-allyl-L-cysteine and S-farnesyl-thiosalicylic acid from Sigma-Aldrich; S-farnesyl-L-cysteine methyl ester and N-acetyl-S-farnesyl-L-cysteine from SantaCruz Biotechnology. Detergents were from Anatrace except for Triton X-100 which was from Sigma-Aldrich. The inhibitors farnesyl-*S-*propargyl, farnesyl-*N-*tranylcypromine, and farnesyl-S-fluorene were purchased from WuXi App Tech. Their purities (HPLC at 220 nm) were higher than 99% and their NMR and mass spectra are shown in the Supporting Information. All other commercial chemicals and proteins for assays were purchased from Sigma-Aldrich.

### Phylogenetic inference and ancestral sequence reconstruction

A dataset of 295 sequences from *Metazoa* was collected by performing homology searches using as query the sequences of human PCYOX1 (Q9UHG3) and PCYOX1-like (Q8NBM8) in BLASTp (32). The search was designed to include sequences of each phylum and explore in more depth the Chordata by selecting at least two species with fully sequenced genomes from each class/subphylum. The multiple sequence alignment was constructed with MAFFTv7 (33). N-terminal signaling peptides and single-sequence extensions were manually trimmed from the multiple-sequence-alignment. The substitution model was selected based on ProtTest v3.4 results (34). Phylogenies were generated employing the maximum likelihood method in RAxMLv8.2.10 with 500 bootstraps (35). Transfer bootstrap expectation (TBE) was calculated with BOOSTER (36). FigTree v1.4.4 was employed to visualize and edit the trees. Ancestral sequence reconstruction was performed as marginal reconstruction in PAMLv4.9 using the codeML module (37). An empirical substitution matrix, empirical equilibrium amino acid frequencies (model = 2), and Jones substitution matrix were used. Four gamma categories were implemented and alpha value was re-inferred. The posterior probability distribution was analyzed at the targeted nodes. Sites were considered ambiguously reconstructed when displaying PP < 0.8 and the second-best state PP > 0.2 (Table S1).

### Cloning and expression

DNA encoding the sequence for mammalian ancestor’s PCYOX1 (26-502; Fig. S2) and PCYOX1-like (23-494) without a signal peptide and containing a six histidine N-terminal affinity tag, were purchased from GenScript and inserted in a pET-24a (+). *E. coli* BL21 RP Plus cells were transformed with the heat-shock method (25 s, 42 °C). The transformed cells were incubated on an LB-agar plate (50 mg/ml chloramphenicol, 25 mg/ml kanamycin) overnight at 37 °C. One colony was picked up from the plate and inoculated in a starter LB media overnight at 37 °C with both antibiotics. The starter culture was then inoculated (1:50) in autoinducing media and growth for 3 hours and then induced at 24 °C for approximately 20 h (PCYOX1) and 37 °C for 4 h (PCYOX1-like). Cells were harvested for 10 min at 5000 *g* (4 °C) and stored at −80 °C.

### Protein production and purification

All procedures were carried out on ice or at 4 °C. The cell pellets (approximately 30 g) were resuspended (1:5) in HEPES 50 mM pH 8, 500 mM NaCl, 100 μM FAD, 0.5 mg/ml lysozyme, 15 mM MgCl_2_, 1 mM phenylmethylsulfonyl fluoride (Sigma Aldrich), 10 μM leupeptin, 10 μM pepstatin (Sigma Aldrich), and 5 μg DNase I per gram of cell paste. The mixture was stirred and incubated at room temperature for 30 min allowing genomic DNA digestion and cell wall lysis. Cells were lysed using a sonicator (5 s on, 5 s off for 5 min, amplitude 40%).

#### PCYOX1

The lysate was incubated with 0.5% (v/v) reduced Triton X-100 overnight at 4 °C for membrane protein extraction. The cell lysate was then centrifuged (56,000 *g*, 1 h, 4 °C). The supernatant was supplemented with 10 mM imidazole and then loaded onto Ni-Resin (GE Healthcare) pre-equilibrated with buffer A (HEPES 25 mM pH 8.0, NaCl 500 mM, 0.2% CHAPS, 5% v/v glycerol, 10 mM imidazole). Proteins were washed with 20 to 30 CV of buffer A and then eluted with 10 CV of buffer B (HEPES 25 mM, NaCl 300 mM, pH 8, 0.2% CHAPS, 5% glycerol, 250 mM imidazole). Protein fractions were concentrated up to 500 μl and loaded onto a Superdex 200 10/300 Gl (GE Healthcare) column pre-equilibrated with gel filtration buffer (25 mM HEPES pH 8.0, 300 mM NaCl, 5% glycerol, and 0.2% CHAPS) and eluted with the same buffer. Fractions containing the enzyme were checked for purity with SDS-PAGE, reunited, concentrated up to 10 mg/ml, and stored at −80 °C. Cofactor retention was monitored with *UV-Vis* spectroscopy. Based on the ratio of the protein (280 nm) and FAD (458 nm) absorbance (A_458_:A_280_ = 1:17), the FAD was fully retained by the protein.

#### PCYOX1-like

Inclusion bodies were pelleted at 10,000 g for 30 min and extracted overnight at 4 °C with 0.5% sodium lauryl sarcosinate (Sigma Aldrich). They were centrifuged at 150,000 g for 1 h and filtered with a 0.45 μM filter. The supernatant was supplemented with 10 mM imidazole and then loaded onto Ni-Resin (GE Healthcare) pre-equilibrated with buffer A (HEPES 25 mM pH 8.5, NaCl 500 mM, 0.5% CHAPS, 10% glycerol, 10 mM imidazole). The protein was washed with 30 CV of Buffer A and then eluted with 10 CV of buffer B (HEPES 25 mM pH 8.5, NaCl 300 mM, 0.5% CHAPS, 10% glycerol, 250 mM imidazole). Fractions containing the protein were concentrated up to 500 μl, incubated with 500 μM FAD, and loaded onto a Superdex 200 10/300 Gl (GE Healthcare) pre-equilibrated with gel filtration buffer, (25 mM HEPES pH 8.0, 300 mM NaCl, 5% glycerol, and 0.5% CHAPS) and eluted with the same buffer. Fractions containing the enzyme were checked with SDS-PAGE, reunited, and stored at −80 °C. The UV-Vis spectrum indicated that the FAD was efficiently incorporated by the protein (A_458_:A_280_ = 1:14).

### Activity and steady-state kinetics assays

Assays were performed in duplicates (n = 2) on a plate reader (ClarioStar, BMG Labtech) at 25 °C. Enzyme activities were measured at different substrate concentrations with an Amplex Red/HRP coupled assay; 0.3 μM of protein were incubated with 0.015 mg/ml HRP, 25 μM of Amplex Red in 25 mM HEPES pH 8.0, 300 mM NaCl, 5% glycerol, 0.2% CHAPS and 5% of DMSO used for the substrate solubilization. The fluorescence of resorufin was detected using an excitation wavelength of 563 nm and an emission wavelength of 587 nm. Data were analyzed using GraphPad Prism 5.

### Pre-steady state kinetics

Fast flavin spectral changes were monitored using a SX20 stopped-flow spectrophotometer equipped with a photomultiplier tube and a photodiode array detector (Applied Photophysics, Surrey, UK). PCYOX1 in 25 mM HEPES pH 8.0, 300 mM NaCl, 5% glycerol, 0.2% CHAPS was used at 12.5 μM final concentration (after mixing). Reactions were run in duplicates by mixing 50 μl of each solution at 25 °C. For both oxidative and reductive half-reactions, PCYOX1 solutions were made anaerobic by flushing the vial with nitrogen for 10 min 5 mM of glucose and 0.3 μM of glucose oxidase (*Aspergillus niger*, type VII, Sigma-Aldrich) were added to the solution to remove the leftover of oxygen, continuing the nitrogen flux for another 5 min. To monitor the reductive half-reaction. PCYOX1 was mixed with three concentrations of *S-*farnesyl-L-cysteine (62.5, 125 and 250 μM, after mixing) (Fig. S9*A*). The 458 nm single-wavelength absorbance data were fitted with a two exponential decay function of SX Pro-Data Viewer software (equation: *a*e^−k1.t^ + *b*e^−k2.t^ + *c*). The first constant (k_fast_) contributed to the major decrease in absorbance, and the second one (k_slow_) to less than 10% in absorbance decrease. To monitor the oxidative half-reaction, PCYOX1 was reduced with 1.5 equivalent of fresh sodium dithionite (Sigma Aldrich) until the yellow color of the solution was completely bleached. *k*_ox_ was determined with three final concentrations of oxygen (0.13, 0.26, and 0.62 mM) (Fig. S9*B*). The single wavelength absorbance data were fitted with a single exponential and slope function of SX Pro-Data Viewer software (equation: *a*e^−k1.t^ + *b*t + *c*). Data were further analyzed and plotted using GraphPad Prism 5.

### GC-MS

Farnesal detection was monitored with GC-MS after the overnight incubation at 30 °C of 1.0 μM PCYOX1 and 1.0 mM salirasib in 25 mM HEPES pH 8.0, 300 mM NaCl, 5% glycerol, and 0.2% CHAPS. 200 μl of reaction mixture were then treated with an equivalent volume of ethyl acetate, vortexed, and harvested at 16,000 *g* for 10 min. The organic phase was subjected to GC separation (Splitless injection, T. Injection = 250 °C, 45 °C for 2 min, temperature gradient=10 °C/min until 300 °C) with Rxi-5ms Cap. Column (30 m, 0.25 mm ID, 0.25 μm) and MS analysis (Positive mode, Full scan=50–650 m/z; EI-quadrupole). Analysis of farnesal was executed by comparing it with the NIST database.

### Thermal shift assay

10 μl of a solution 20 μM PCYOX1 or PCYOX1-like in gel filtration buffer were run on a Tycho NT.6 system in Tycho capillaries (both by NanoTemper Technologies; capillaries cat# TY-C001) to analyze protein quality and induce thermal stabilization with a 10-fold excess of ligand/substrate. The Tycho technology, a modified version of nanoDSF (nano Differential Scanning Fluorimetry), runs a thermal ramp from 35 °C to 95 °C at a defined rate of 30 °C/min and records intrinsic protein fluorescence from tryptophan and tyrosine residues at 330 nm and 350 nm throughout the run. The data are automatically analyzed by the system's software to calculate inflection temperatures (Ti), a measure for the temperature(s) at which the protein unfolds. Controls are run with 5% of DMSO.

### IC_50_ and K_i_ determination

#### IC_50_ determination

0.3 μM of PCYOX1 in 25 mM HEPES pH 8.0, 300 mM NaCl, 5% glycerol, 0.2% CHAPS were incubated for 10 min with different concentrations of DMSO-dissolved *N-*farnesyl-tranylcypromine or *S-*farnesyl-propargyl. Enzymatic activities were measured through the addition of 20 μM *S-*farnesyl-L-cysteine using the Amplex Red/HRP assay. A control containing only 5% DMSO, and no inhibitor was used to define 100% of enzymatic activity. Fluorescence data were fitted in Graphpad 5.0 with a log (inhibitor) *vs* response (three-parameter) equation.

#### K_i_ determination

Michaelis-Menten curves were registered after the incubation of 0.3 μM of PCYOX1 with different concentrations of *N-*farnesyl-tranylcypromine or *S-*farnesyl-propargyl. Data were fitted in GraphPad 5.0 with competitive or non-competitive inhibition equations.

### Microscale thermophoresis

PCYOX1 was labelled with RED-NHS second generation (Lysine Residues). 20 nM labelled enzymes were titrated with different concentrations of salirasib ranging from 1 mM to 300 nM. Fluorescence data were fitted with the following equation:

$$f\left(c_{ligand}\right)=Unbound+\left(Bound-Unbound\right)\frac{c_{ligand}+c_{target}+K_{d}-\sqrt{{\left(c_{ligand}+c_{target}+K_{d}\right)}^{2}-4c_{ligand}c_{target}}}{2c\;target}$$

where *f(c*_*ligand*_*)* is the F_norm_ value at a given ligand concentration c_ligand_; *Unbound* is the F_norm_ signal of the target alone; *Bound* is the F_norm_ signal of the complex; *K*_*d*_ is the dissociation constant or binding affinity; and **c**_**target**_ is the final concentration of target in the assay.

### Crystallization and structural determination of PCYOX1

CYOX1, 10 mg/ml in 25 mM HEPES pH 8.0, 300 mM NaCl, 5% glycerol, 0.2% CHAPS, was crystallized by the hanging drop technique using 1.8 to 2 M ammonium sulfate, 0.1 M HEPES pH 6.5 and 5% isopropanol as reservoir solution. Crystals appeared after 3 days at 20 °C. Data were collected at the European Synchrotron Radiation Facility (Grenoble, France) and integrated with XDS (38). Data reduction was carried out with Aimless (39) and data analysis with Xtriage (40). The phase problem was solved by molecular replacement with Phaser (41) using as search models the Alphafold2 predicted structure (21). Refinement was carried out with REFMAC (42) and model building with COOT (43). Figures were generated with PyMOL 2.0.

## Data availability

Mammalian ancestors PCYOX1 and PCYOX1-like have been deposited under the Genbank accession codes: PP883551 and PP883552 respectively. Crystal structure of ancestral PCYOX1 has been deposited under PDB, accession code: 9FXQ.

## Supporting information

This article contains supporting information.

## Conflict of interest

The authors declare that they have no competing interests with the content of this article.

## References

[1] Jiang H., Zhang X., Chen X., Aramsangtienchai P., Tong Z., Lin H. (2018). Protein lipidation: occurrence, mechanisms, biological functions, and enabling Technologies. Chem. Rev..

[2] Casey P.J. (1992). Biochemistry of protein prenylation. J. Lipid Res..

[3] Wang M., Casey P.J. (2016). Protein prenylation: unique fats make their mark on biology. Nat. Rev. Mol. Cell. Biol..

[4] Winter-Vann A.M., Casey P.J. (2005). Post-prenylation-processing enzymes as new targets in oncogenesis. Nat. Rev. Cancer.

[5] Tate E.W., Soday L., de la Lastra A.L., Wang M., Lin H. (2024). Protein lipidation in cancer: mechanisms, dysregulation and emerging drug targets. Nat. Rev. Cancer.

[6] Zhang L., Tschantz W.R., Casey P.J. (1997). Isolation and characterization of a prenylcysteine lyase from bovine brain. J. Biol. Chem..

[7] Tschantz W.R., Zhang L., Casey P.J. (1999). Cloning, expression, and cellular localization of a human prenylcysteine lyase. J. Biol. Chem..

[8] Tschantz W.R., Digits J.A., Pyun H.J., Coates R.M., Casey P.J. (2001). Lysosomal prenylcysteine lyase is a FAD-dependent thioether oxidase. J. Biol. Chem..

[9] Beigneux A., Withycombe S.K., Digits J.A., Tschantz W.R., Weinbaum C.A., Griffey S.M. (2002). Prenylcysteine lyase deficiency in mice results in the accumulation of farnesylcysteine and geranylgeranylcysteine in brain and liver. J. Biol. Chem..

[10] Banfi C., Brioschi M., Barcella S., Wait R., Begum S., Galli S. (2009). Proteomic analysis of human low-density lipoprotein reveals the presence of prenylcysteine lyase, a hydrogen peroxide-generating enzyme. Proteomics.

[11] Banfi C., Baetta R., Barbieri S.S., Brioschi M., Guarino A., Ghilardi S. (2021). Prenylcysteine oxidase 1, an emerging player in atherosclerosis. Commun. Biol..

[12] Petenkova A., Auger S.A., Lamb J., Quellier D., Carter C., To O.T. (2023). Prenylcysteine oxidase 1 like protein is required for neutrophil bactericidal activities. Nat. Commun..

[13] Nicoll C.R., Massari M., Fraaije M.W., Mascotti M.L., Mattevi A. (2023). Impact of ancestral sequence reconstruction on mechanistic and structural enzymology. Curr. Opin. Struct. Biol..

[14] Kumar S., Suleski M., Craig J.M.-, Kasprowicz A.E., Sanderford M., Li M. (2022). TimeTree 5: an expanded resource for species divergence times. Mol. Biol. Evol..

[15] Randall R.N., Radford C.E., Roof K.A., Natarajan D.K., Gaucher E.A. (2016). An experimental phylogeny to benchmark ancestral sequence reconstruction. Nat. Commun..

[16] Chisnall B., Johnson C., Kulaberoglu Y., Chen Y.W. (2014). Insoluble protein purification with sarkosyl: facts and precautions. Methods Mol. Biol..

[17] Huizinga H., Denton R., Koehler K.G., Tomasello A., Wood L., Sen S.E. (2010). Farnesylcysteine lyase is involved in negative regulation of abscisic acid signaling in Arabidopsis. Mol. Plant.

[18] Digits J.A., Pyun H.J., Coates R.M., Casey P.J. (2002). Stereospecificity and kinetic mechanism of human prenylcysteine lyase, an unusual thioether oxidase. J. Biol. Chem..

[19] van Kempen M., Kim S.S., Tumescheit C., Mirdita M., Lee J., Gilchrist C.L.M. (2024). Fast and accurate protein structure search with Foldseek. Nat. Biotechnol..

[20] Gupta A., Dong A., Lowe A.W. (2012). AGR2 gene function requires a unique endoplasmic reticulum localization motif. J. Biol. Chem..

[21] Abramson J., Adler J., Dunger J., Evans R., Green T., Pritzel A. (2024). Accurate structure prediction of biomolecular interactions with AlphaFold 3. Nature.

[22] Binda C., Li M., Hubalek F., Restelli N., Edmondson D.E., Mattevi A. (2003). Insights into the mode of inhibition of human mitochondrial monoamine oxidase B from high-resolution crystal structures. Proc. Natl. Acad. Sci. U. S. A..

[23] Iacovino L.G., Reis J., Mai A., Binda C., Mattevi A. (2020). Diphenylene Iodonium is a noncovalent MAO inhibitor: a biochemical and structural analysis. ChemMedChem.

[24] Bustinza-Linares E., Kurzrock R., Tsimberidou A.M. (2010). Salirasib in the treatment of pancreatic cancer. Future Oncol..

[25] Forneris F., Mattevi A. (2008). Enzymes without borders: mobilizing substrates, delivering products. Science.

[26] Suazo K.F., Bělíček J., Schey G.L., Auger S.A., Petre A.M., Li L. (2023). Thinking outside the CaaX-box: an unusual reversible prenylation on ALDH9A1. RSC Chem. Biol..

[27] Mayoral J.G., Nouzova M., Navare A., Noriega F.G. (2009). NADP+-dependent farnesol dehydrogenase, a corpora allata enzyme involved in juvenile hormone synthesis. Proc. Natl. Acad. Sci. U. S. A..

[28] Fraser J.S., Murcko M.A. (2024). Structure is beauty, but not always truth. Cell.

[29] Uhlén M., Fagerberg L., Hallström B.M., Lindskog C., Oksvold P., Mardinoglu A. (2015). Proteomics. Tissue-based map of the human proteome. Science.

[30] Thul P.J., Åkesson L., Wiking M., Mahdessian D., Geladaki A., Ait Blal H. (2017). A subcellular map of the human proteome. Science.

[31] Maurer-Stroh S., Koranda M., Benetka W., Schneider G., Sirota F.L., Eisenhaber F. (2007). Towards complete sets of farnesylated and geranylgeranylated proteins. PLoS Comput. Biol..

[32] Camacho C., Boratyn G.M., Joukov V., Vera Alvarez R., Madden T.L. (2023). ElasticBLAST: accelerating sequence search via cloud computing. BMC Bioinformatics.

[33] Katoh K., Standley D.M. (2013). MAFFT multiple sequence alignment software version 7: improvements in performance and usability. Mol. Biol. Evol..

[34] Darriba D., Taboada G.L., Doallo R., Posada D. (2011). ProtTest 3: fast selection of best-fit models of protein evolution. Bioinformatics.

[35] Stamatakis A. (2006). RAxML-VI-HPC: maximum likelihood-based phylogenetic analyses with thousands of taxa and mixed models. Bioinformatics.

[36] Lemoine F., Domelevo Entfellner J.B., Wilkinson E., Correia D., Dávila Felipe.M., De Oliveira T. (2018). Renewing Felsenstein's phylogenetic bootstrap in the era of big data. Nature.

[37] Yang Z. (2007). Paml 4: phylogenetic analysis by maximum likelihood. Mol. Biol. Evol..

[38] Kabsch W. (2010). Xds. Acta Crystallogr. D Biol. Crystallogr..

[39] Agirre J., Atanasova M., Bagdonas H., Ballard C.B., Baslé A., Beilsten-Edmands J. (2023). The CCP4 suite: integrative software for macromolecular crystallography. Acta Crystallogr. D Biol. Crystallogr..

[40] Liebschner D., Afonine P.V., Baker M.L., Bunkóczi G., Chen V.B., Croll T.I. (2019). Macromolecular structure determination using X-rays, neutrons and electrons: recent developments in Phenix. Acta Crystallogr. D Biol. Crystallogr..

[41] McCoy A.J., Grosse-Kunstleve R.W., Adams P.D., Winn M.D., Storoni L.C., Read R.J. (2007). Phaser crystallographic software. J. Appl. Crystallogr..

[42] Vagin A.A., Steiner R.A., Lebedev A.A., Potterton L., McNicholas S., Long F. (2004). REFMAC5 dictionary: organization of prior chemical knowledge and guidelines for its use. Acta Crystallogr. D Biol. Crystallogr..

[43] Emsley P., Cowtan K. (2004). Coot: model-building tools for molecular graphics. Acta Crystallogr. D Biol. Crystallogr..

[44] Chen R., Jiang X., Sun D., Han G., Wang F., Ye M. (2009). Glycoproteomics analysis of human liver tissue by combination of multiple enzyme digestion and hydrazide chemistry. J. Proteome Res..

[45] Liu T., Qian W.J., Gritsenko M.A., Camp D.G., Monroe M.E., Moore R.J. (2005). Human plasma N-glycoproteome analysis by immunoaffinity subtraction, hydrazide chemistry, and mass spectrometry. J. Proteome Res..

[46] Ochoa D., Jarnuczak A.F., Viéitez C., Gehre M., Soucheray M., Mateus A. (2020). The functional landscape of the human phosphoproteome. Nat. Biotechnol..

[47] Corso G., Stärk H., Jing B., Barzilay R., Jaakkola T. (2022). DiffDock: diffusion steps, twists, and turns for molecular docking. arXiv.

