# Supplementary material for: Evolution, structure, and drug-metabolizing activity of mammalian prenylcysteine oxidases
Source: J Biol Chem. 2024 Sep 24;300(11):107810. doi: 10.1016/j.jbc.2024.107810 (PMC11530802; doi:10.1016/j.jbc.2024.107810)
Supplement: Supplemental Tables and Figures [file mmc2.pdf]

Evolution, Structure, and Drug-Metabolising Activity of Mammalian Prenylcysteine Oxidases

Marco Barone[1], Letizia Pizzorni[1], Marco W. Fraaije[2], Maria L. Mascotti[3*], Andrea Mattevi[1*]

[1]Department of Biology and Biotechnology "Lazzaro Spallanzani", University of Pavia, Pavia, Italy 27100.

[2]Molecular Enzymology Group, University of Groningen, Groningen, The Netherlands, 9747AG.

[3]IHEM CONICET, Universidad Nacional de Cuyo, Mendoza, Argentina, M5502JMA.

***Correspondence**: mlmascotti@mendoza-conicet.gob.ar; andrea.mattevi@unipv.it

File content:

Figures S1 to S9

Tables S1 to S2

NMR and Mass Spectrometry characterisation of the PCYOX inhibitors

Other supporting materials for this manuscript:

Dataset S1

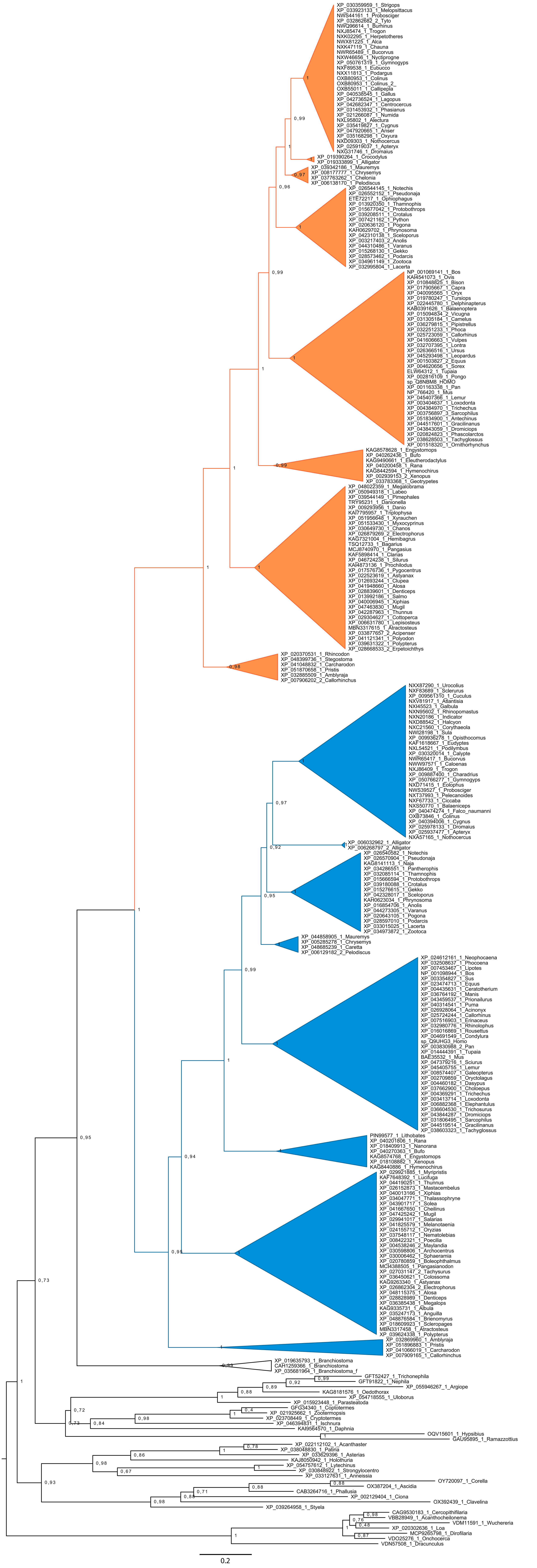
XP_030359959_1_Strigops
XP_033923133_1_Melopsittacus
NWS44161_1_Probosciger
XP_032862682_2_Tyto
NWQ96614_1_Burhinus
NXJ85474_1_Trogon
NXK02295_1_Herpetotheres
NWX81225_1_Alca
NXK47119_1_Chauna
NWR65489_1_Bucorvus
NXW46656_1_Nyctiprogne
XP_050761319_1_Gymnogyps
NXF89538_1_Eubucco
NXX11813_1_Podargus
OXB80953_1_Colinus
OXB80953_1_Colinus_2_
OXB55011_1_Callipepla
XP_040538545_1_Gallus
XP_042736524_1_Lagopus
XP_042682347_1_Centrocercus
XP_031453932_1_Phasianus
XP_021266087_1_Numida
NXL95802_1_Alectura
XP_035419827_1_Cygnus
XP_047920665_1_Anser
XP_035168298_1_Oxyura
NXD09303_1_Nothocercus
XP_025919037_1_Apteryx
NXG31746_1_Dromaius
XP_019390264_1_Crocodylus
XP_019333899_1_Alligator
XP_039342186_1_Mauremys
XP_008177777_1_Chrysemys
XP_037763262_1_Chelonia
XP_006138170_1_Pelodiscus
XP_026544145_1_Notechis
XP_026552152_1_Pseudonaja
ETE72217_1_Ophiophagus
XP_013920350_1_Thamnophis
XP_015677042_1_Protobothrops
XP_039208511_1_Crotalus
XP_007421162_1_Python
XP_020636120_1_Pogona
KAH0629702_1_Phrynosoma
XP_042310138_1_Sceloporus
XP_003217403_2_Anolis
XP_044310486_1_Varanus
XP_015268130_1_Gekko
XP_028573462_1_Podarcis
XP_034961149_1_Zootoca
XP_032995804_1_Lacerta
NP_001069141_1_Bos
KAI4541073_1_Ovis
XP_010848825_1_Bison
XP_017905667_1_Capra
XP_040095565_1_Oryx
XP_019780247_1_Tursiops
XP_022445780_1_Delphinapterus
KAB0391626_1_Balaenoptera
XP_015094834_2_Vicugna
XP_031305184_1_Camelus
XP_036279815_1_Pipistrellus
XP_032251233_1_Phoca
XP_025723059_1_Callorhinus
XP_041606663_1_Vulpes
XP_032707395_1_Lontra
XP_026366516_1_Ursus
XP_045293498_1_Leopardus
XP_001503827_2_Equus
XP_004620656_1_Sorex
ELW64312_1_Tupaia
XP_002816109_1_Pongo
sp_Q8NBM8_HOMO
XP_001163338_1_Pan
NP_766420_1_Mus
XP_045407366_1_Lemur
XP_003404637_1_Loxodonta
XP_004384970_1_Trichechus
XP_003756897_3_Sarcophilus
XP_051834900_1_Antechinus
XP_044517601_1_Gracilinanus
XP_043843059_1_Dromiciops
XP_020824823_1_Phascolarctos
XP_038628503_1_Tachyglossus
XP_001518320_1_Ornithorhynchus
KAG8578628_1_Engystomops
XP_040262436_1_Bufo
KAG9490661_1_Eleutherodactylus
XP_040200458_1_Rana
KAG8442594_1_Hymenochirus
XP_002939153_2_Xenopus
XP_033783368_1_Geotrypetes
XP_048022359_1_Megalobrama
XP_050949318_1_Labeo
XP_039544149_1_Pimephales
TRY95231_1_Danionella
XP_009293956_1_Danio
KAI7795957_1_Triplophysa
XP_051956648_1_Xyrauchen
XP_051533430_1_Myxocyprinus
XP_030649730_1_Chanos
XP_026879269_2_Electrophorus
KAG7321004_1_Hemibagrus
TSQ12733_1_Bagarius
MCJ8740970_1_Pangasius
KAF5898414_1_Clarias
XP_046724238_1_Silurus
KAI4873136_1_Prochilodus
XP_017576736_1_Pygocentrus
XP_022523619_1_Astyanax
XP_012693244_1_Clupea
XP_041948660_1_Alosa
XP_028839601_1_Denticeps
XP_013992186_1_Salmo
XP_040006945_1_Xiphias
XP_047463830_1_Mugil
XP_042287963_1_Thunnus
XP_029304627_1_Cottoperca
XP_006631780_1_Lepisosteus
MBN3317615_1_Atractosteus
XP_033877657_2_Acipenser
XP_041121341_1_Polyodon
XP_039631322_1_Polypterus
XP_028668533_2_Erpetoichthys
XP_020370531_1_Rhincodon
XP_048399736_1_Stegostoma
XP_041048832_1_Carcharodon
XP_051870658_1_Pristis
XP_032885509_1_Amblyraja
XP_007906202_2_Callorhinchus
NXX87290_1_Urocolius
NXF83689_1_Sclerurus
XP_009561310_1_Cuculus
NXV81917_1_Atlantisia
NXI45523_1_Galbula
NXN95602_1_Rhinopomastus
NXN20186_1_Indicator
NXD88542_1_Halcyon
NXC21560_1_Corythaeola
NWI28198_1_Sula
XP_009936278_1_Opisthocomus
KAF1618667_1_Eudyptes
NXL54521_1_Podilymbus
XP_030320014_1_Calypte
NWR65417_1_Bucorvus
NWW97571_1_Caloenas
NXJ86409_1_Trogon
XP_009887400_1_Charadrius
XP_050766277_1_Gymnogyps
NXD71415_1_Eolophus
NWS39527_1_Probosciger
NXT37993_1_Pelecanoides
NXF67733_1_Ciccaba
NXS50770_1_Balaeniceps
XP_040474274_1_Falco_naumanni
OXB73846_1_Colinus
XP_040394008_1_Cygnus
XP_025978133_1_Dromaius
XP_025937477_1_Apteryx
NXA57165_1_Nothocercus
XP_006032962_1_Alligator
XP_006268797_2_Alligator
XP_026540582_1_Notechis
XP_026570904_1_Pseudonaja
KAG8141113_1_Naja
XP_034286551_1_Pantherophis
XP_032085114_1_Thamnophis
XP_015666594_1_Protobothrops
XP_039180088_1_Crotalus
XP_015276615_1_Gekko
XP_042328017_1_Sceloporus
KAH0623034_1_Phrynosoma
XP_016854706_1_Anolis
XP_044273305_1_Varanus
XP_020643105_1_Pogona
XP_028597010_1_Podarcis
XP_033015025_1_Lacerta
XP_034973872_1_Zootoca
XP_044858905_1_Mauremys
XP_005285278_1_Chrysemys
XP_048685239_1_Caretta
XP_006129182_2_Pelodiscus
XP_024612161_1_Neophocaena
XP_032508637_1_Phocoena
XP_007453467_1_Lipotes
NP_001098944_1_Bos
XP_003354827_1_Sus
XP_023474713_1_Equus
XP_004435631_1_Ceratotherium
XP_036764192_1_Manis
XP_043459537_1_Prionailurus
XP_040314541_1_Puma
XP_026928064_1_Acinonyx
XP_025724244_1_Callorhinus
XP_007516903_1_Erinaceus
XP_032980776_1_Rhinolophus
XP_016016869_1_Rousettus
XP_004691549_1_Condylura
sp_Q9UHG3_Homo
XP_003830988_2_Pan
XP_014444391_1_Tupaia
BAE35532_1_Mus
XP_047379216_1_Sciurus
XP_045405755_1_Lemur
XP_008574407_1_Galeopterus
XP_002709859_1_Oryctolagus
XP_004460182_1_Dasypus
XP_037662900_1_Choloepus
XP_004369291_1_Trichechus
XP_003413714_1_Loxodonta
XP_006882368_1_Elephantulus
XP_036604530_1_Trichosurus
XP_043844287_1_Dromiciops
XP_031806495_1_Sarcophilus
XP_044519514_1_Gracilinanus
XP_038603323_1_Tachyglossus
PIN99577_1_Lithobates
XP_040201806_1_Rana
XP_018409913_1_Nanorana
XP_040270363_1_Bufo
KAG8574768_1_Engystomops
XP_018108882_1_Xenopus
KAG8440886_1_Hymenochirus
XP_029921885_1_Myripristis
KAF7648392_1_Lucifuga
XP_044190251_1_Thunnus
XP_026152873_1_Mastacembelus
XP_040013166_1_Xiphias
XP_034047771_1_Thalassophryne
XP_043901717_1_Solea
XP_041667650_1_Cheilinus
XP_047425242_1_Mugil
XP_029941017_1_Salarias
XP_041825579_1_Melanotaenia
XP_024155712_1_Oryzias
XP_037548117_1_Nematolebias
XP_008422321_1_Poecilia
XP_004538246_2_Maylandia
XP_030598806_1_Archocentrus
XP_030006462_1_Sphaeramia
XP_020780859_1_Boleophthalmus
MCI4388505_1_Pangasianodon
XP_027031147_2_Tachysurus
XP_036450621_1_Colossoma
KAG9263340_1_Astyanax
XP_026862304_2_Electrophorus
XP_048115375_1_Alosa
XP_028828989_1_Denticeps
XP_036385438_1_Megalops
KAG9335731_1_Albula
XP_035247173_1_Anguilla
XP_048876584_1_Brienomyrus
XP_018609923_1_Scleropages
MBN3317458_1_Atractosteus
XP_039624338_1_Polypterus
XP_032869960_1_Amblyraja
XP_051896883_1_Pristis
XP_041066019_1_Carcharodon
XP_007909165_1_Callorhinchus
XP_019635793_1_Branchiostoma
CAH1259366_1_Branchiostoma
XP_035681964_1_Branchiostoma_f
GFT52427_1_Trichonephila
GFT91822_1_Nephila
XP_055946267_1_Argiope
KAG8181576_1_Oedothorax
XP_054718555_1_Uloborus
XP_015923448_1_Parasteatoda
GFG34340_1_Coptotermes
XP_021925662_1_Zootermopsis
XP_023708449_1_Cryptotermes
XP_046394831_1_Ischnura
KAI9564570_1_Daphnia
OQV15601_1_Hypsibius
GAU95895_1_Ramazzottius
XP_022112102_1_Acanthaster
XP_038048830_1_Patiria
XP_033629396_1_Asterias
KAJ8050942_1_Holothuria
XP_054757612_1_Lytechinus
XP_030848922_1_Strongylocentro
XP_033127631_1_Anneissia
OY720097_1_Corella
OX387204_1_Ascidia
CAB3264716_1_Phallusia
XP_002129404_1_Ciona
OX392439_1_Clavelina
XP_039264958_1_Styela
CAG9530183_1_Cercopithifilaria
VBB28949_1_Acanthocheilonema
VDM11591_1_Wuchereria
XP_020302636_1_Loa
MCP9265798_1_Dirofilaria
VDO25276_1_Onchocerca
VDN57508_1_Dracunculus
0.2

Fig. S1. Annotated phylogeny of chordata PCYOXs. The tree was inferred by RAxMLv8.2.10 with 500 bootstraps. The multiple sequence alignment included 295 sequences and 483 sites. TBE values are shown at the nodes. Accession codes and species names are given for each sequence. Clades in orange correspond to the PCYOX1 paralogue and in blue to PCYOX1-like. Scale bar indicates substitutions per site.

>PCYOX1 from mammalian ancestor

RAPPDKIAVIGAGIGGTSAAYYLRQKFGKDVQIDVFERGEVGGRLATLNVEGQEYEAGGSVIHPL
NLHMKRFVKELGLSVSQGAGGLMGIYNGETFVFEESSWFIINVIKLLWHYGFQFLRMHMWVEDIL
DKFMRIYRYQSHDYAFSSVEGLLHALGGDEFTRMLNRTILEALQKAGFSQKFLNEIVTPAMRVNY
GQSTNINGFVGAVSLAGADSGLWSVEGGNKLVCSGLLQASKARLISGSVTSIEEKTRTKQTGGTV
KLYEVTYQTDSGTASDLYDIVVVATPLNRKMSNITFLNFNPPIPEFHKYYHQTVTTFVHGRLNAS
FFGYKAPDSFHLTDILTTDNPDLFINSIGVVSPVKEKADAASPPATGSAVWKVFSQEPLTKEQLN
LLFASYDSVKEKKWLAYPHYKPPEKCPPIILHDRMYYLNGIEWAASAMEMSAIAAHNAALLAYHR
WYGNTEMIDQEGLYEKLKTEL

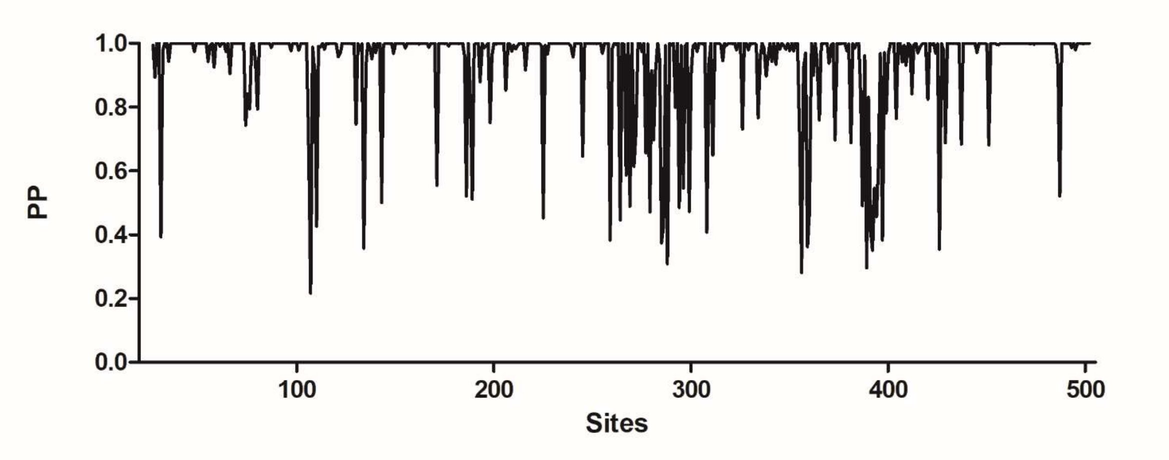

Overall PP=0.93

>**PCYOX1-like from mammalian ancestor**

DAPPGKIAVVGAGIGGSAVAHFLQQHFGPQVQIDVYEKGTVGGRLATITVNKQQYESGGASFHSL
NLHMQDFVKLLGLKHRREVAGKSAIFGGEQFVLEETDWYLLNLFRLWWHYGISFLRLQMWVEEVM
EKFMRIYKYQAHGYAFSGVEELLHSLGGDTFVNMTQRSVAESLLEVGVTQRFIDDVIAAVLRASY
GQSVSMPAFAGAMSLAGAQGNLWAVEGGNKLVCSGLLKLTKANVIHARVTAVALHSSEGKALYQV
RYENEAGQGSDFYDMVVIATPLHPGSNSNITFEGFDPPIPEFLGSFQPTVTSLVHGYLNSSYFGF
PDPKLFPFASILTTDSPSLFFCSLDNICPVNISATFRRKQPQEAAVWRVLSPQPLERPQLKTLFR
SYYSVQTAEWQAHPHYGSRATLPPFALHDQLFYLNGLEWAASSVEMSAVAAKNVALLAYNRWYQD
LEKIDQKDLMHKVKTEL

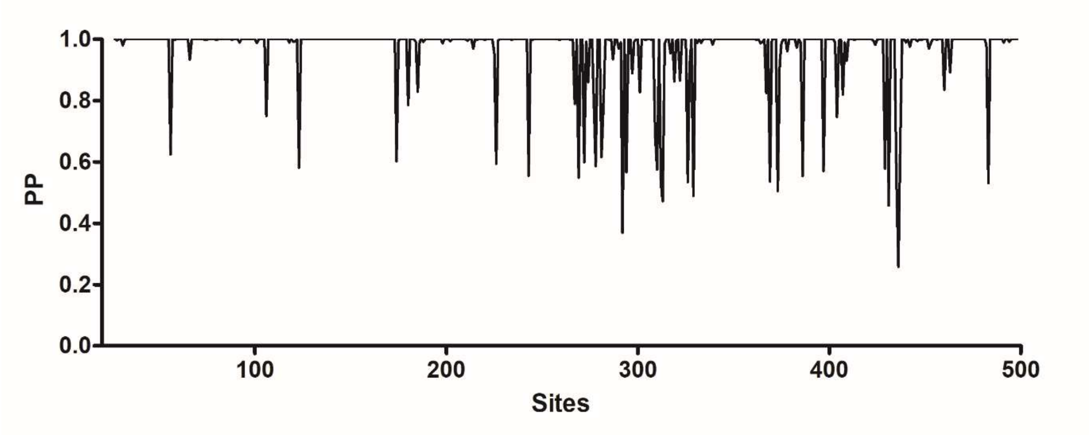

Overall PP=0.96

Fig. S2. Amino acid sequences of the reconstructed prenylcysteine oxidases used in our work. They do not comprise their respective N-terminal signalling peptide. The graphs show the posterior probability distribution per site of the reconstructed sequences.

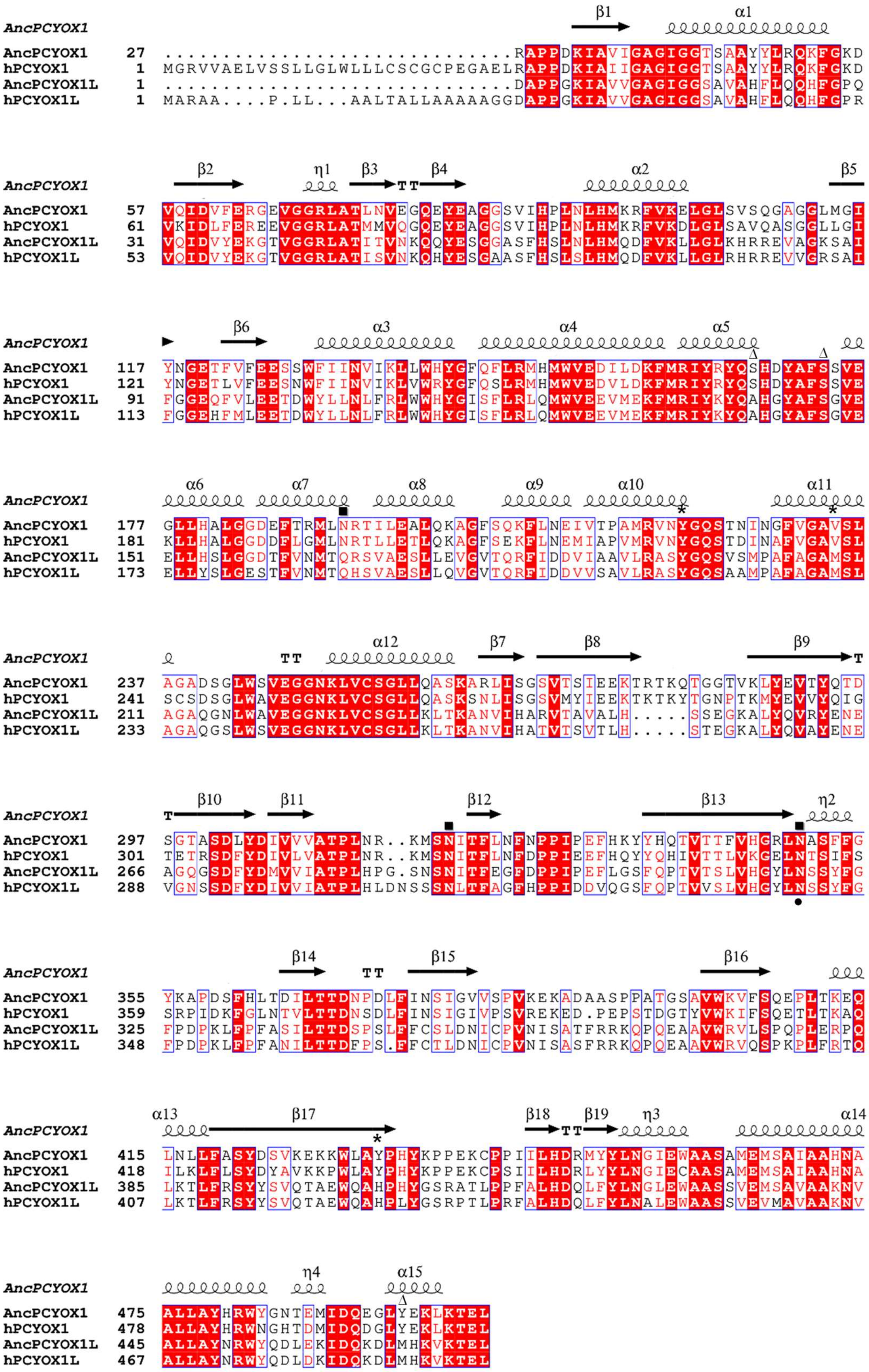

Fig. S3. Multiple sequence alignment of mammalian ancestral PCYOX1 (AncPCYOX1), human PCYOX1 (hPCYOX1), mammalian ancestral PCYOX1-like (AncPCYOX1L), and human PCYOX1-like (hPCYOX1L). Mammalian ancestral PCYOX1 was numbered in accordance with the crystal structure. An asterisk (*) marks residues of mammalian ancestral PCYOX1 subjected to

mutagenesis (Y221A, V234W and Y433A). An empty triangle (Δ) denotes human PCYOX1 sites of phosphorylation (S171, S177 and Y498; conserved in mammalian ancestral PCYOX1, PP=1). A black square (▪) marks human PCYOX1 sites of N-glycosylation (N196, N323, N353, conserved in mammalian ancestral PCYOX1; PP=1). A black circle (●) marks the only N-glycosylation of human PCYOX1L (N342), conserved in AncPCYOX1L with a PP=1.

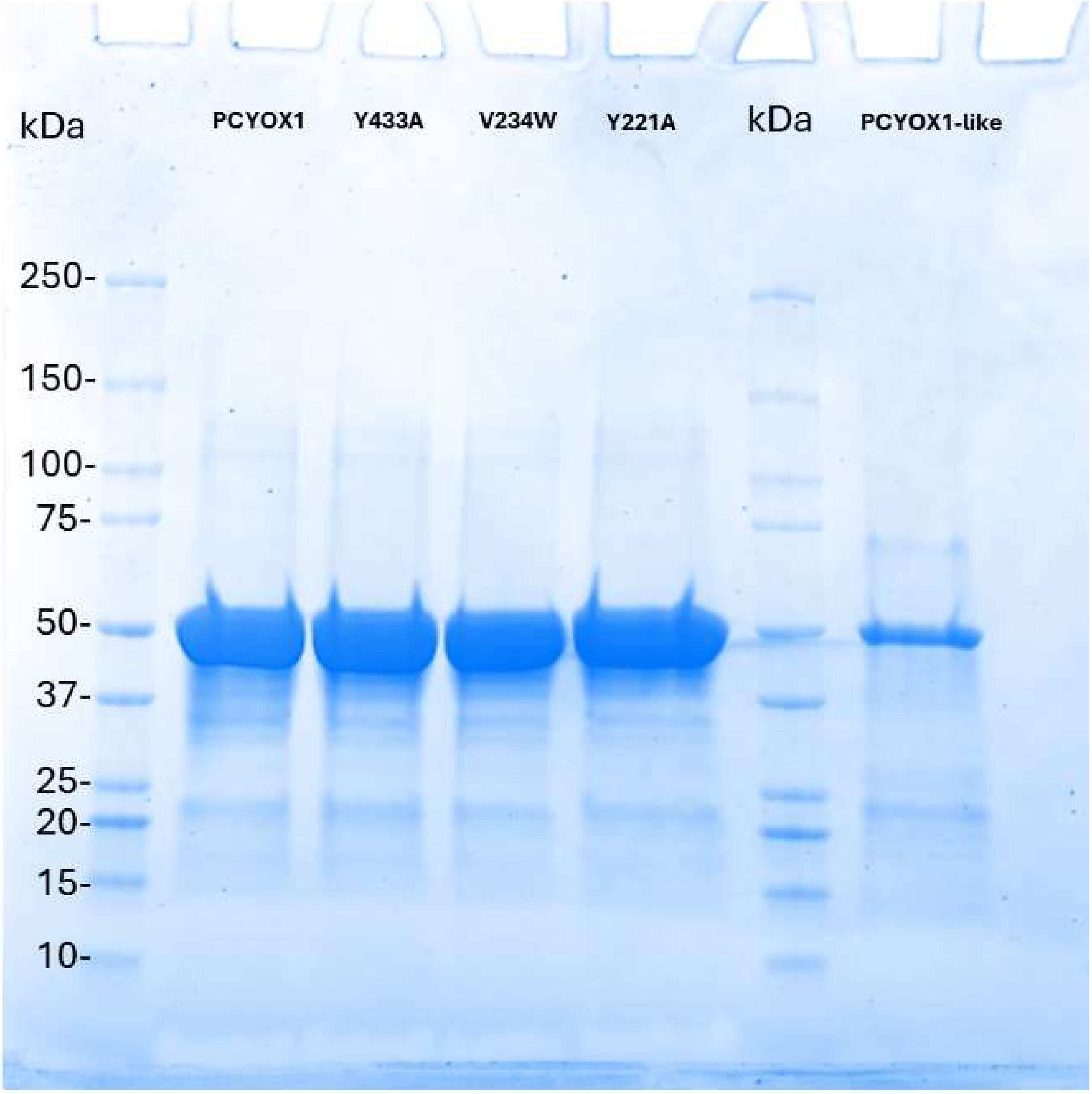

Fig. S4. SDS-PAGE of purified wild-type and mutant PCYOX1, and PCYOX1-like proteins. The gels are Coomassie stained.

a

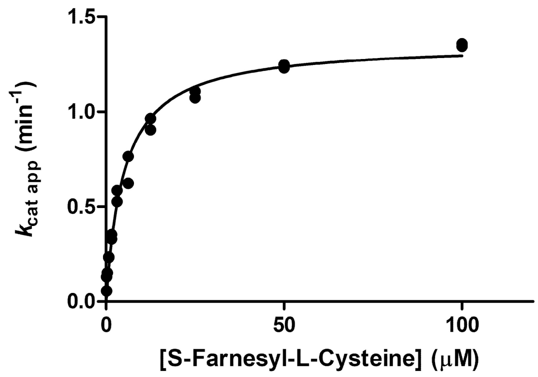

$k_{cat\ app}$ (min^{-1})
1.5
1.0
0.5
0.0
0
50
100
[S-Farnesyl-L-Cysteine] (μM)

b

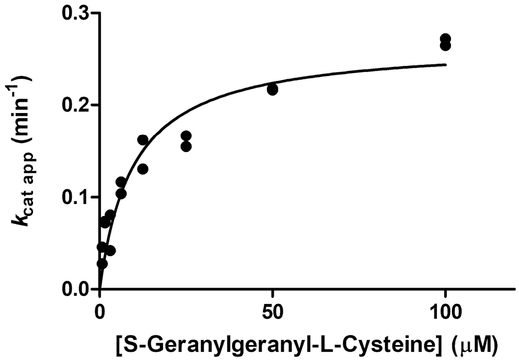

$k_{cat\ app}$ (min^{-1})
0.3
0.2
0.1
0.0
0
50
100
[S-Geranylgeranyl-L-Cysteine] (μM)

c

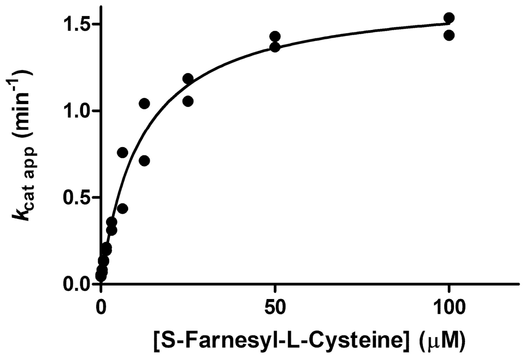

$k_{cat\ app}$ (min^{-1})
1.5
1.0
0.5
0.0
0
50
100
[S-Farnesyl-L-Cysteine] (μM)

d

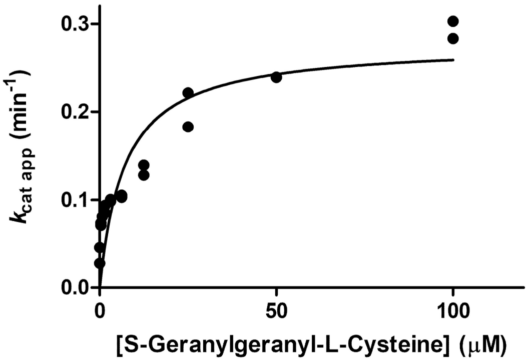

$k_{cat\ app}$ (min^{-1})
0.3
0.2
0.1
0.0
0
50
100
[S-Geranylgeranyl-L-Cysteine] (μM)

e

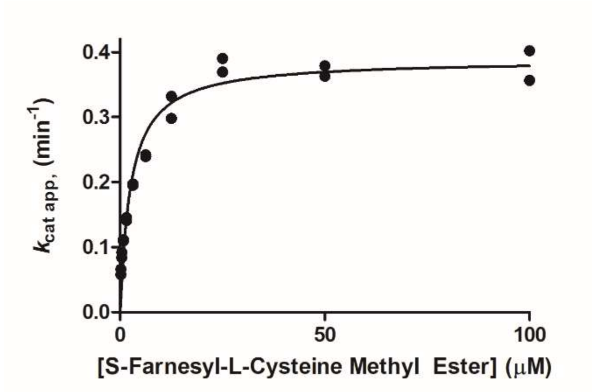

$k_{cat\ app,}$ (min^{-1})
0.4
0.3
0.2
0.1
0.0
0
50
100
[S-Farnesyl-L-Cysteine Methyl Ester] (μM)

f

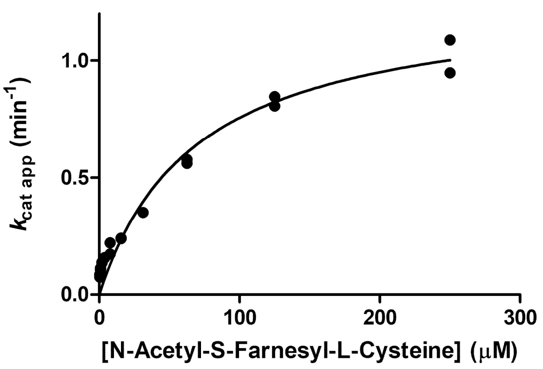

$k_{cat\ app}$ (min^{-1})
1.0
0.5
0.0
0
100
200
300
[N-Acetyl-S-Farnesyl-L-Cysteine] (μM)

g

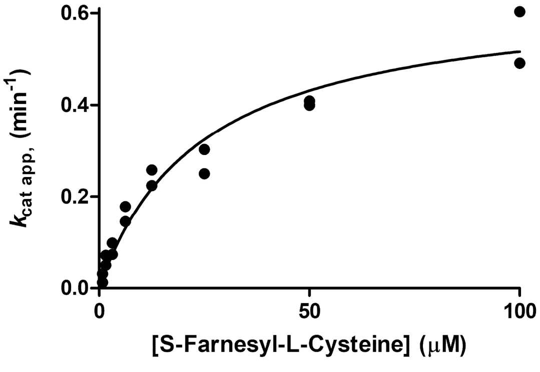

$k_{cat\ app,}$ (min^{-1})
0.6
0.4
0.2
0.0
0
50
100
[S-Farnesyl-L-Cysteine] (μM)

h

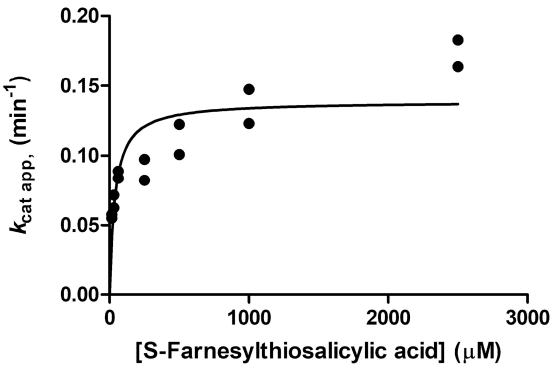

$k_{cat\ app,}$ (min^{-1})
0.20
0.15
0.10
0.05
0.00
0
1000
2000
3000
[S-Farnesylthiosalicylic acid] (μM)

Fig. S5. Steady-state kinetics of PCYOX1 and PCYOX1-like enzymes. (a) PCYOX1 with *S*-farnesyl-L-cysteine. **(b)** PCYOX1 with *S*-geranylgeranyl-L-cysteine. **(c)** PCYOX1-like with *S*-farnesyl-L-cysteine. **(d)** PCYOX1-like with *S*-geranylgeranyl-L-cysteine. **(e)** PCYOX1 with *S*-farnesyl-L-cysteine O-methyl ester. **(f)** PCYOX1 with *N*-acetyl-*S*-farnesyl-L-cysteine. **(g)** PCYOX1 Y433A with *S*-farnesyl-L-cysteine. **(h)** PCYOX1 with *S*-farnesyl- thiosalicylic acid (salirasib). n = 2 independent experiments, individually plotted as dots. Each panel illustrates the fitting of the data to the Michaelis-Menten curve.

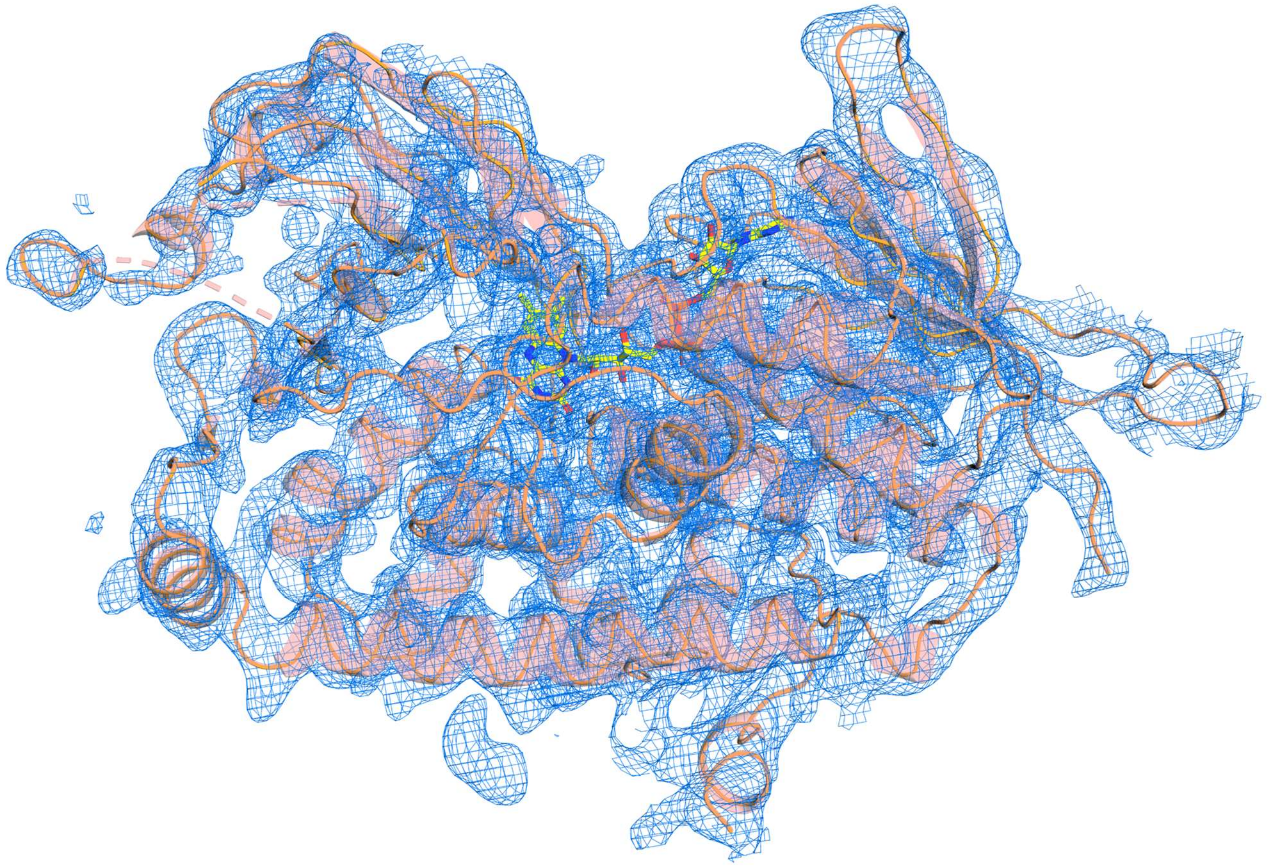

Fig. S6. Crystallographic data. Electron density of the PCYOX1 monomer. The weighted 2Fo-Fc map is contoured at 1.2 σ and was calculated right after molecular replacement executed using ligand-free structures as search models.

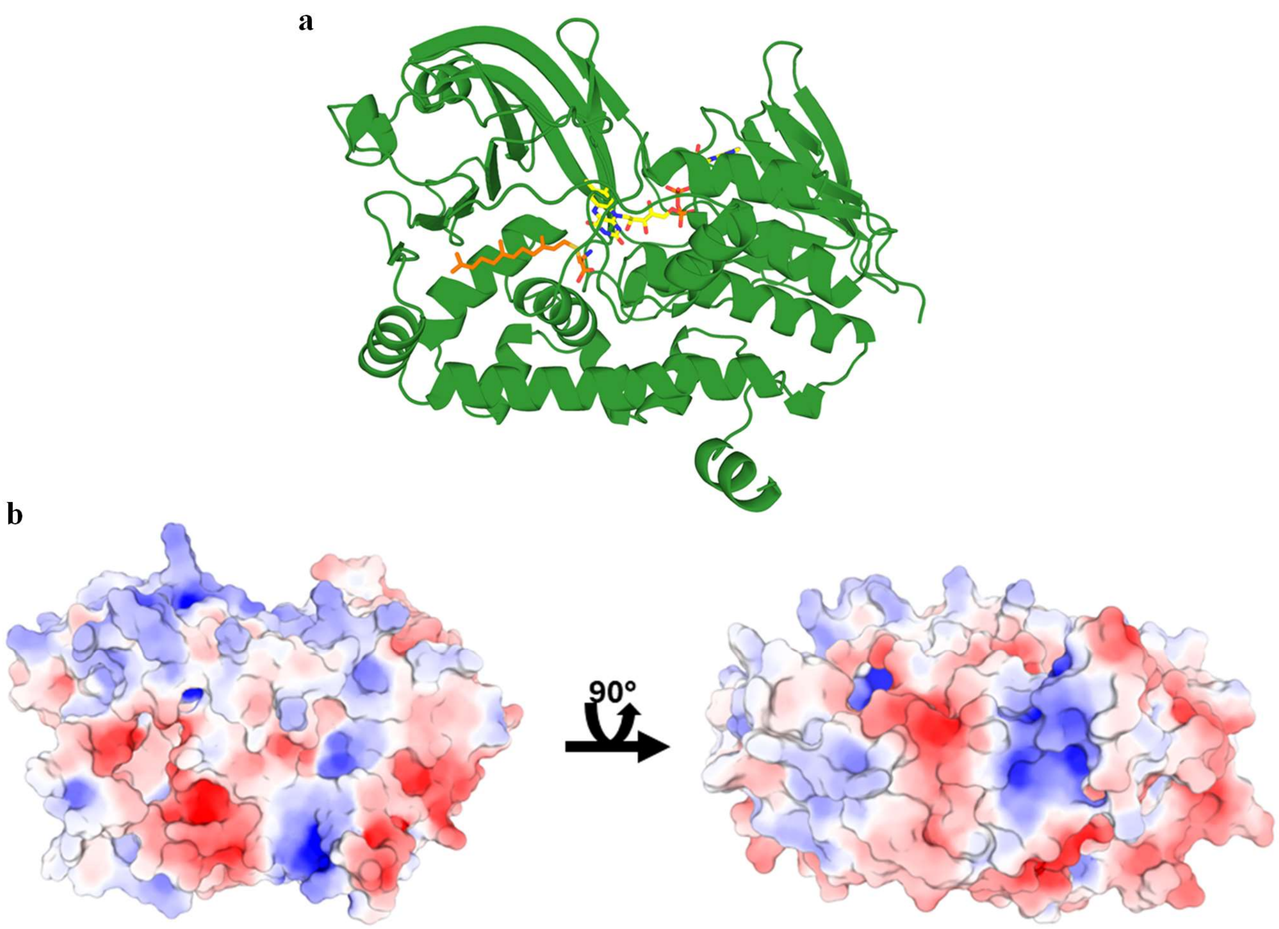

Fig. S7. The Alphafold3 predicted structure of PCYOX1-like. **(a)** The overall structure with the predicted binding of FAD and S-farnesyl-L-cysteine. **(b)** Electrostatic potential surface of PCYOX1-like.

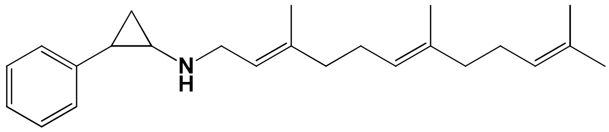

a

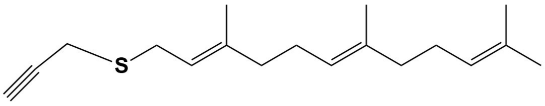

Farnesyl-*N*-Tranylcypromine **Farnesyl-*S*-Propargyl**

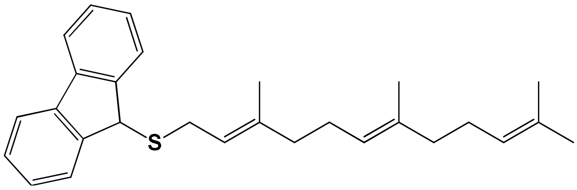

Farnesyl-*S*-Fluorene

b

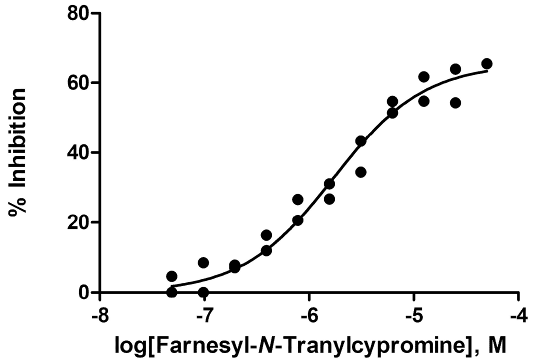

c

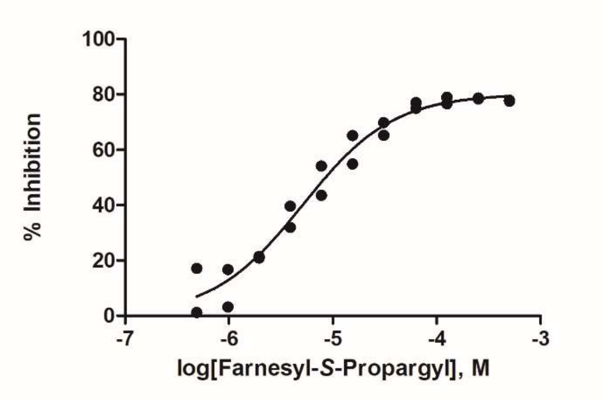

d

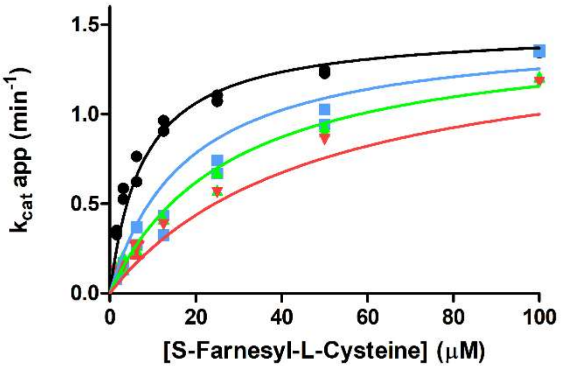

e

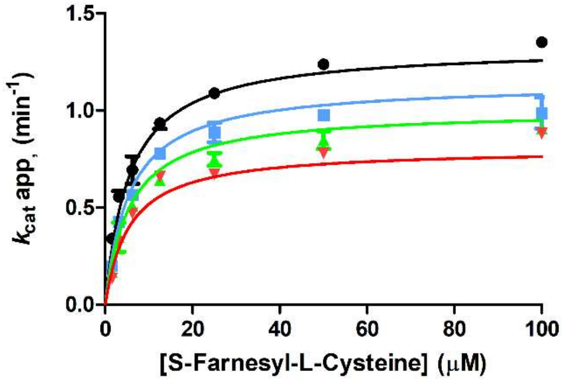

Fig. S8. Inhibitor design. (a) Compounds tested in inhibition studies. **(b-c)** IC_{50} determination for farnesyl-N-tranylcypromine (**IC_{50}=2.2±1.1 μM**) and farnesyl-S-propargyl (**IC_{50}=5.1±1.2 μM**) at 20 μM substrate concentration. (**d-e**) K_i determination for farnesyl-N-tranylcypromine (K_i=613±95 nM) and farnesyl-S-propargyl (K_i=4.82±0.42 μM). The black line represents the uninhibited protein. Blue, green and red lines represent experiments at 780 nM, 1560 nM, and 3125 nM inhibitor concentrations. n = 2 independent experiments, individually plotted as dots.

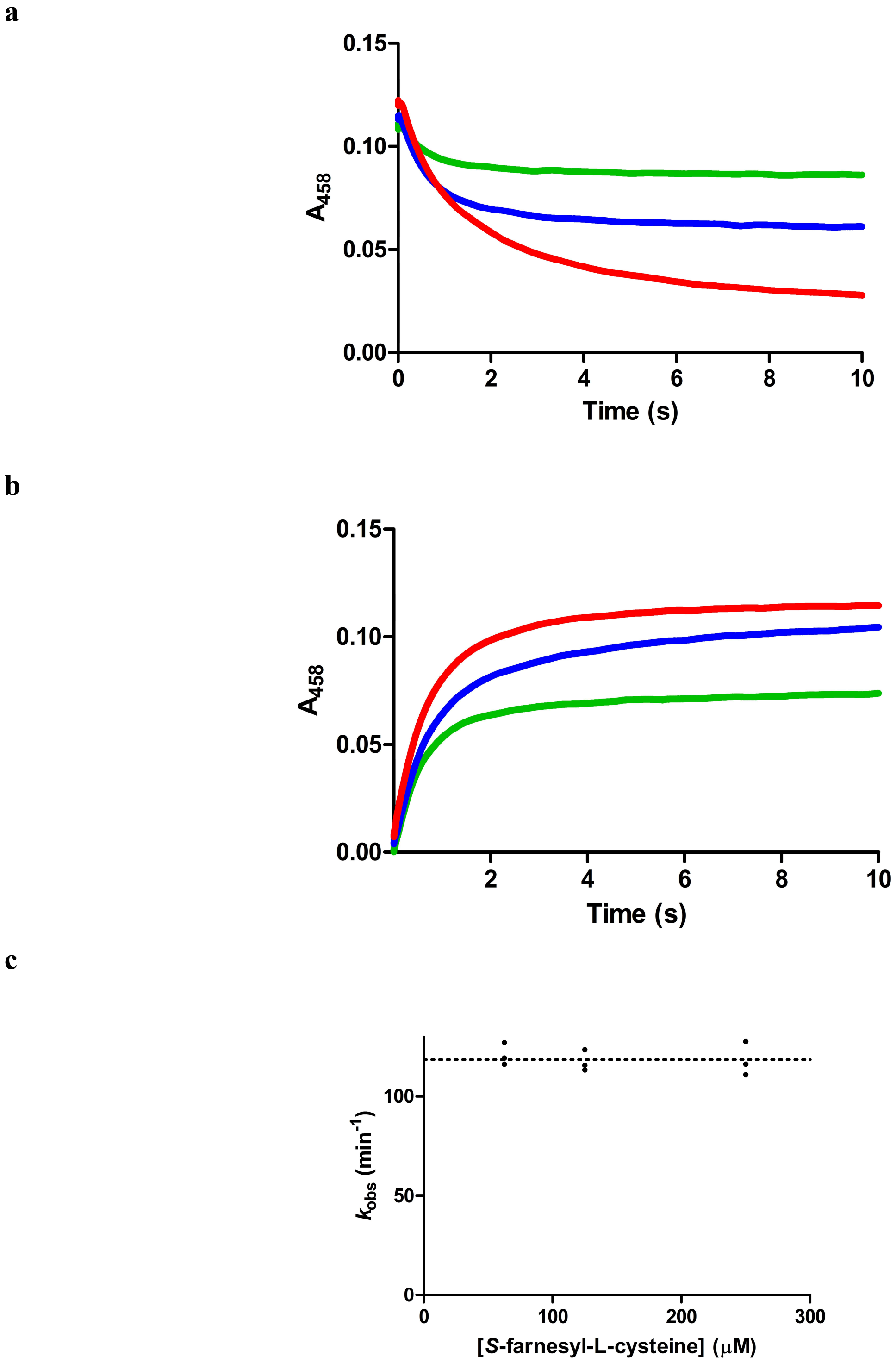

Fig. S9. Pre steady-state kinetics of PCYOX1. (a) Traces for the reductive half-reaction of 12.5 μM oxidized PCYOX1 (after mixing) with three *S*-farnesyl-L-cysteine concentrations (62.5, 125 and 250 μM, in green, blue and red). Each trace is the average of three replicates. **(b)** Traces for the oxidative half-reaction of 12.5 μM dithionite-reduced PCYOX1 (after mixing) with three oxygen concentrations (0.13, 0.31 and 0.62 mM, in green, blue and red). Each line is the average of three replicates. **(c)** Observed rates for flavin reduction after mixing the oxidized enzyme (12.5 μM) with *S*-farnesyl-l-cysteine (62.5, 125 and 250 μM, in green, blue and red line). The dotted line represents the observed k_{red} (118.7 min^{-1}).

Table S1. Posterior probabilities of the ambiguously reconstructed sites of mammalian ancestor's PCYOX1 and PCYOX1-like

PCYOX1				
Site	map Anc	PP	Alt Anc	PP
31	D	0.39	N	0.29
80	E	0.79	D	0.21
107	S	0.22	P	0.21
110	A	0.43	T	0.23
130	F	0.75	Y	0.26
143	F	0.50	L	0.49
171	A	0.55	S	0.44
186	E	0.52	D	0.47
189	R	0.51	G	0.25
225	T	0.45	A	0.36
245	S	0.64	A	0.35
259	Q	0.38	Y	0.22
264	R	0.44	Q	0.29
267	S	0.58	P	0.27
269	S	0.48	T	0.37
271	T	0.61	M	0.23
277	T	0.65	A	0.31
279	T	0.47	S	0.26
281	Q	0.69	R	0.20
285	T	0.37	P	0.32
286	V	0.43	T	0.20
288	L	0.30	S	0.23
294	Q	0.48	E	0.23
296	D	0.54	A	0.22
308	V	0.40	L	0.34
311	T	0.64	A	0.35
326	N	0.73	D	0.22
356	K	0.28	Q	0.27
359	D	0.36	G	0.29
360	S	0.46	P	0.28
365	D	0.75	E	0.23
381	V	0.68	A	0.23
387	E	0.49	D	0.21
389	A	0.29	P	0.27
392	A	0.35	Q	0.33
394	P	0.45	K	0.43
397	T	0.38	A	0.33
404	V	0.76	I	0.23
426	K	0.35	R	0.33
437	K	0.68	R	0.22
487	E	0.52	D	0.47

PCYOX1-like				
Site	map Anc	PP	Alt Anc	PP
52	Q	0.62	R	0.37
102	H	0.75	Q	0.25
170	G	0.60	S	0.23
222	S	0.59	T	0.37
268	A	0.59	S	0.34
273	S	0.78	N	0.22
274	S	0.58	T	0.39
277	K	0.62	R	0.38
288	A	0.36	E	0.34
290	Q	0.57	H	0.28
305	H	0.68	Y	0.31
308	S	0.52	G	0.34
309	N	0.47	D	0.30
322	P	0.50	A	0.21
325	I	0.49	P	0.35
365	S	0.53	N	0.37
369	C	0.51	R	0.28
382	T	0.55	A	0.30
393	V	0.57	I	0.43
425	H	0.58	Y	0.42
427	H	0.46	Q	0.33
431	R	0.54	Q	0.29
432	A	0.26	S	0.25
479	E	0.53	D	0.46

map= maximum a posteriori

Table S2. Crystallographic data collection and refinement statistics

	**PCYOX1**
PDB code	9FXQ
Resolution range	122.1-3.10 (3.31-3.10)
Space group	P $4_1$32
Unit cell axes (Å)	172.66 172.66 172.66
Unique reflections	16624 (2936)
Multiplicity	57.6 (26.7)
Completeness (%)	100 (100)
Mean I/sigma(I)	15.3 (1.56)
R-merge	0.18 (6.48)
$CC_{1/2}$	1.00 (0.34)
R-work	0.24
R-free	0.28
N° non-hydrogen atoms	3729
ligands	75
Protein residues	464
RMS bonds (Å)	0.46
RMS angles (°)	0.89
Ramachandran favoured (%)	93
Ramachandran allowed (%)	7
Ramachandran outliers (%)	0.2

NMR and Mass Spectrometry characterisation of the PCYOX inhibitors

Farnesyl-N-tranylcypromine

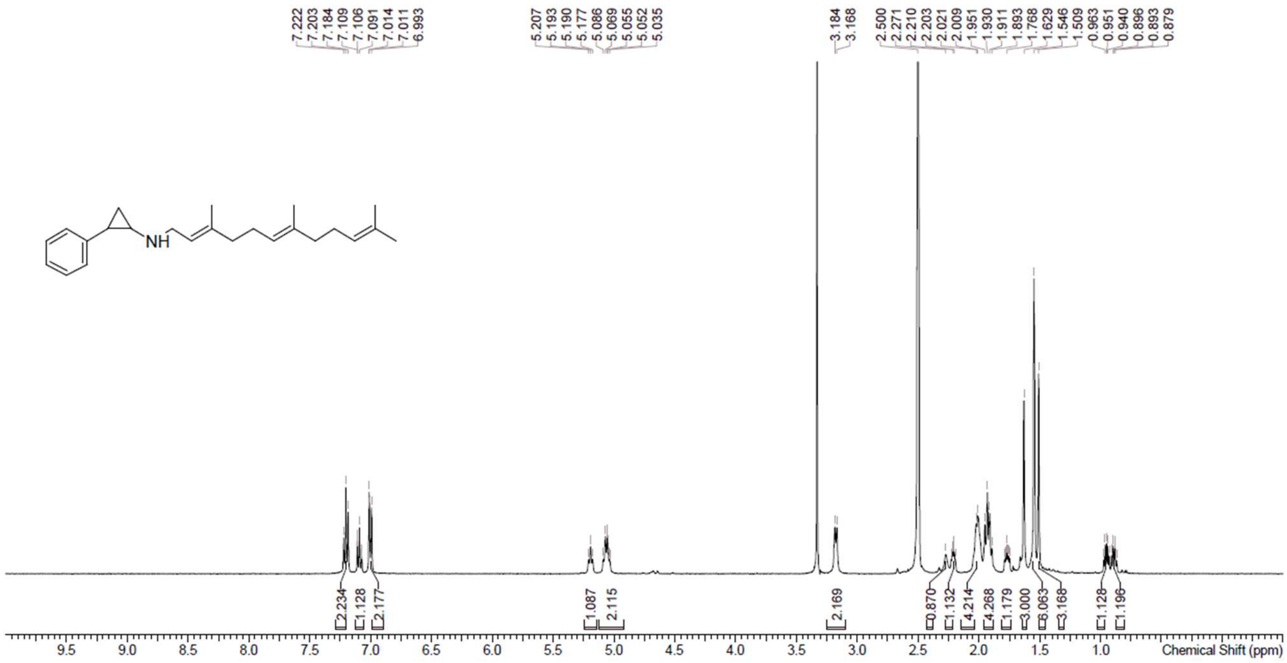

^{1}H NMR: DMSO-d_6, 400MHz

δ 7.25 - 7.16 (m, 2H), 7.12 - 7.06 (m, 1H), 7.04 - 6.95 (m, 2H), 5.25 - 5.14 (m, 1H), 5.13 - 4.92 (m, 2H), 3.18 (br d, J = 6.4 Hz, 2H), 2.27 (br s, 1H), 2.24 - 2.18 (m, 1H), 2.02 (br d, J = 4.8 Hz, 4H), 1.96 - 1.88 (m, 4H), 1.77 (ddd, J = 3.6, 5.6, 8.8 Hz, 1H), 1.63 (s, 3H), 1.55 (s, 6H), 1.51 (s, 3H), 0.95 (td, J = 4.4, 9.2 Hz, 1H), 0.89 (td, J = 5.2, 6.8 Hz, 1H)

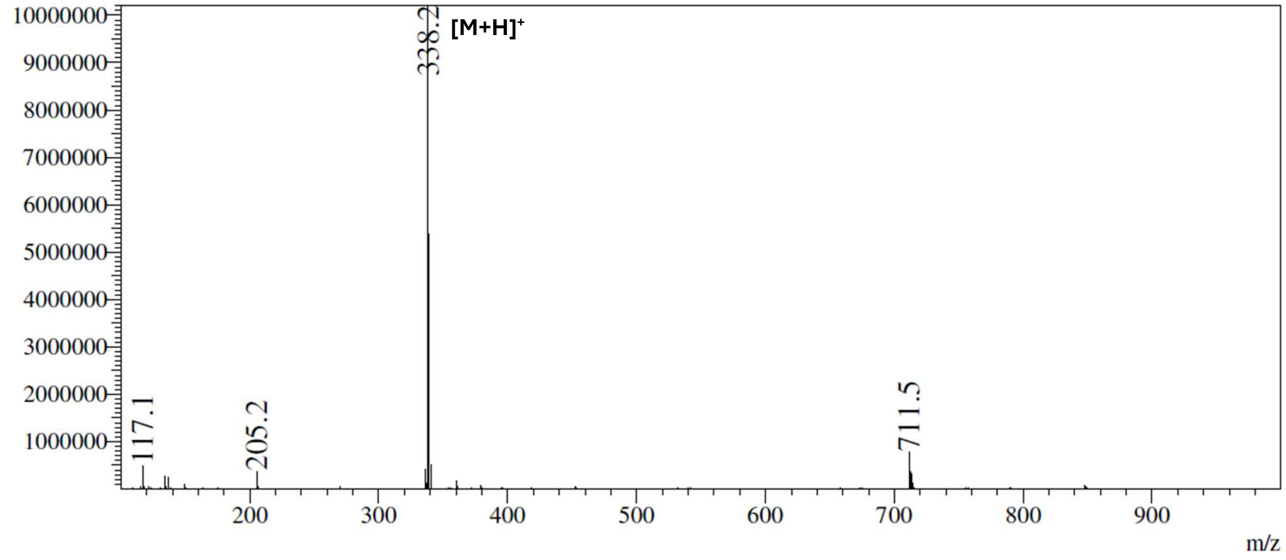

product RT = 1.440 min, MS (ESI) m/z = 338.2 [M+H]$^+$

Farnesyl-S-propargyl

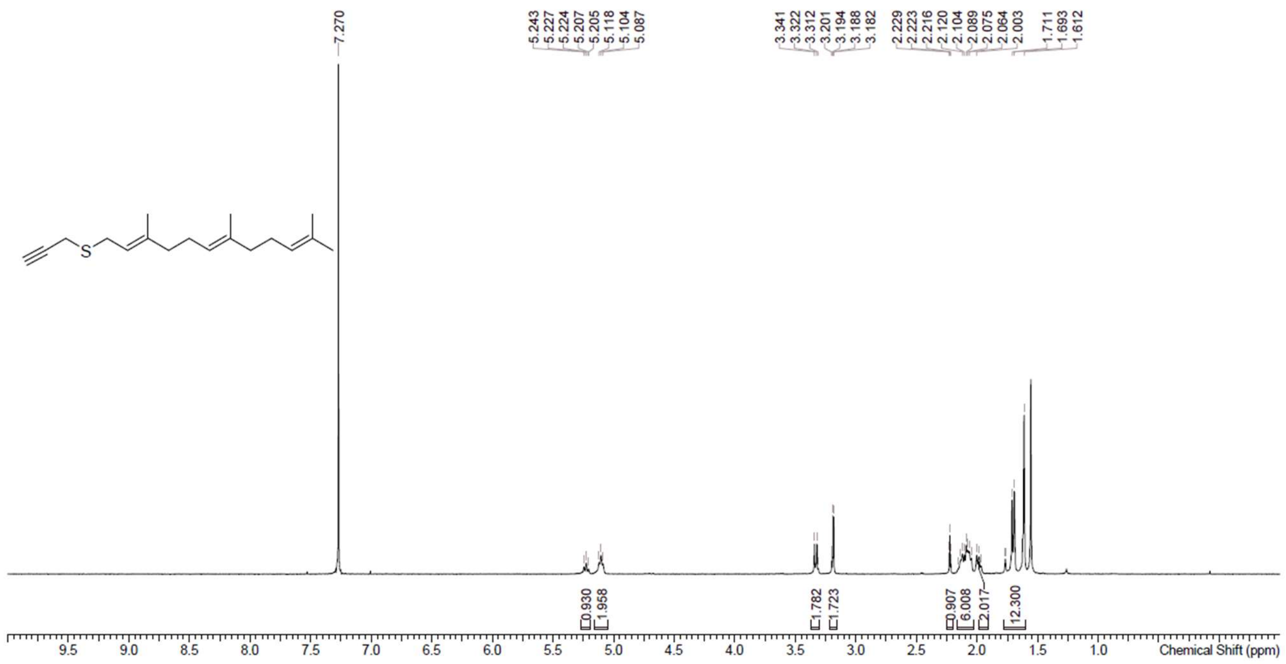

^{1}H NMR: $CDCl_3$, 400MHz

δ 5.27 - 5.19 (m, 1H), 5.15 - 5.05 (m, 2H), 3.37 - 3.30 (m, 2H), 3.21 - 3.16 (m, 2H), 2.22 (t, J = 2.4 Hz, 1H), 2.17 - 2.03 (m, 6H), 2.02 - 1.95 (m, 2H), 1.78 - 1.67 (m, 6H), 1.61 (s, 6H)

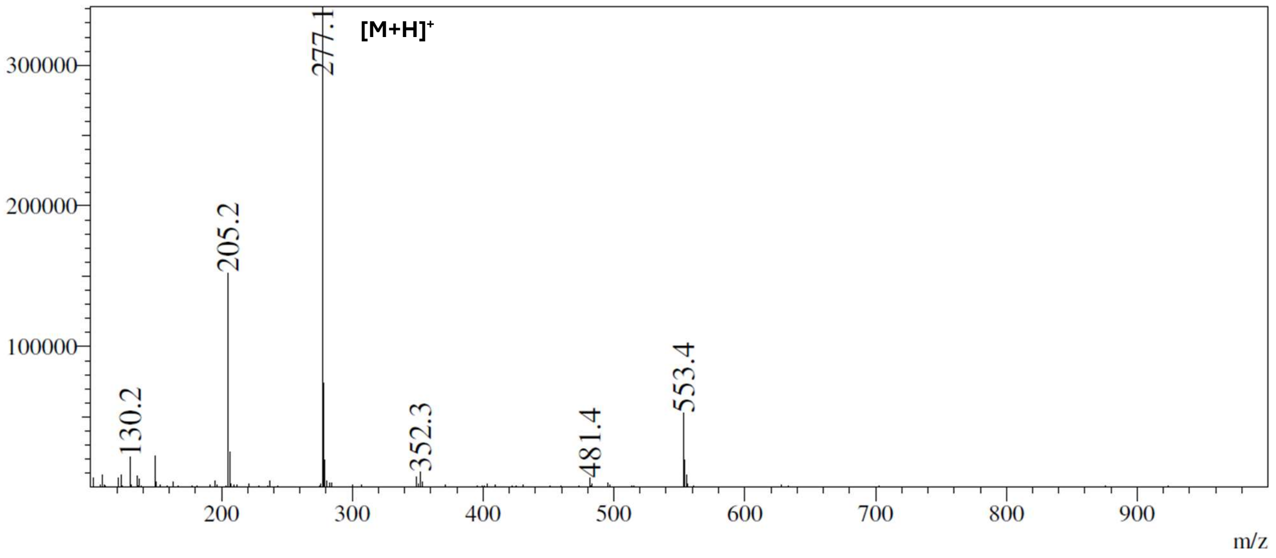

product RT = 1.352 min, MS (ESI) m/z = 277.1 $[M+H]^+$

Farnesyl-S-fluorene

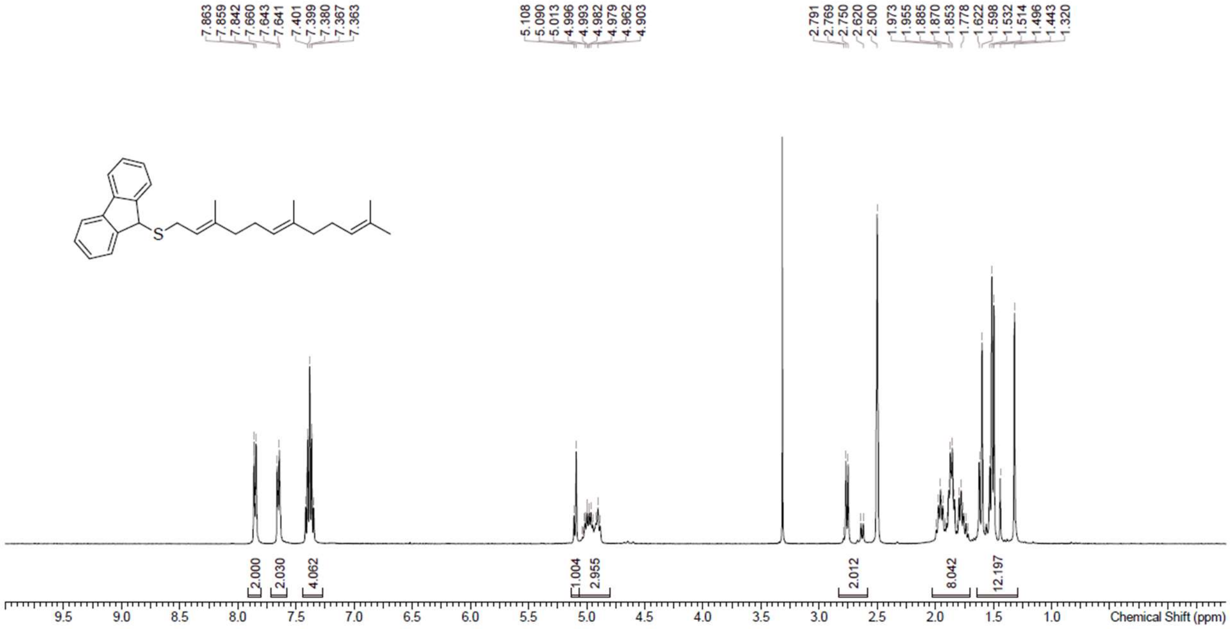

^{1}H NMR: DMSO-d_6, 400MHz

δ 7.91 - 7.80 (m, 2H), 7.71 - 7.58 (m, 2H), 7.44 - 7.27 (m, 4H), 5.13 - 5.07 (m, 1H), 5.07 - 4.80 (m, 3H), 2.83 - 2.59 (m, 2H), 2.03 - 1.70 (m, 8H), 1.64 - 1.29 (m, 12H)

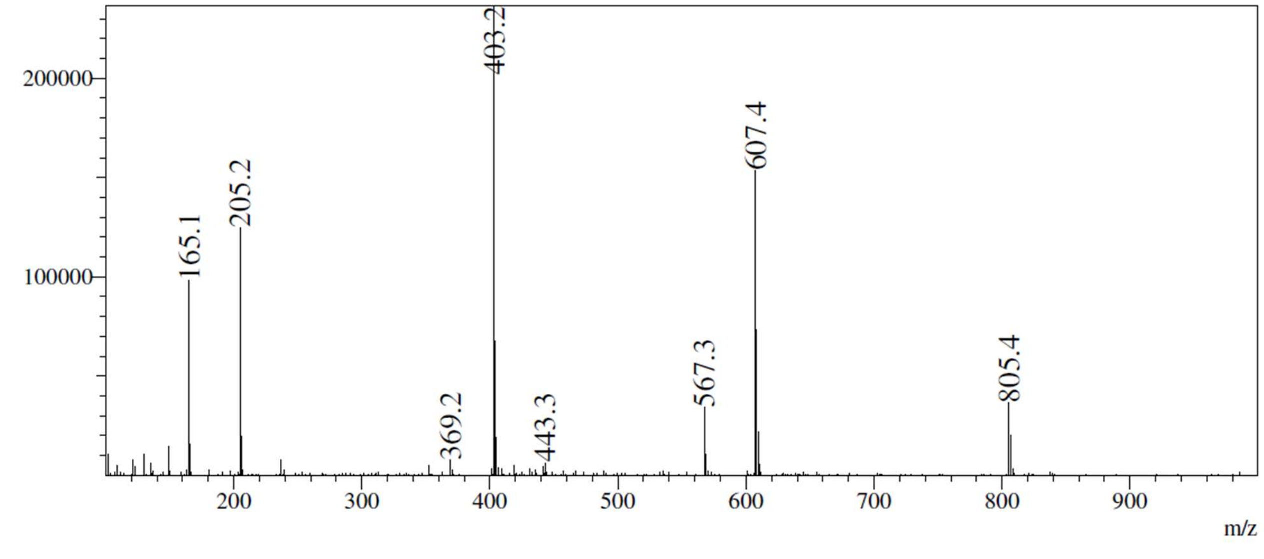

product RT = 1.980 min, MS (ESI) *m/z* = 403.2 $[M+H]^+$

Dataset S1 (separate file). Raw data for Figures 1c-d-e-f-g, 2a-b-c, 2c-d, 4a-b, S2, S5, S8, S9 and Table 1.
